# Grafting redox-active thiols into MOF channels for dual-interfacial regulation in Li–S batteries

**DOI:** 10.1039/d6sc05954f

**Published:** 2026-08-28

**Authors:** Yu Li, Kaiyan Wu, Cheng Zhang, Zhuangzhuang Zhang, Bofang Shi, Chen Liu, Suyue Guo, Zhicheng Zhang, Mingbo Ma, Honghui Yang

**Affiliations:** a Xi'an Key Laboratory of Sustainable Energy Materials Chemistry, School of Chemistry, Xi'an Jiaotong University Xi'an 710049 China yanghonghui@mail.xjtu.edu.cn

## Abstract

Lithium–sulfur batteries (LSBs) deliver exceptional theoretical specific capacities yet suffer severe practical restrictions from polysulfide shuttling and lithium dendrite growth. Although metal–organic frameworks (MOFs) are popular separator modifiers for LSBs, they rarely integrate efficient polysulfide adsorption/catalysis with uniform lithium-ion conduction. Herein, redox-active dithiothreitol (DTT) is grafted onto the inner metal sites of MIL-101(Cr) channels to fabricate functional polypropylene (PP) separators. On the cathode side, DTT cooperates with the MOF scaffold to immobilize polysulfides, where thiol moieties streamline their redox transformation. Meanwhile, grafted DTT installs abundant lithiophilic –SH and –OH polar sites across the MOF interior. Coupled with size sieving from regular MOF pores, these sites equalize Li^+^ flux, trigger steady lithium nucleation/deposition, and effectively suppressed the growth of lithium dendrites. Enabled by dual cathode–anode interfacial modulation, this coating clearly elevates battery capacity and cyclability: Li‖Li batteries sustain stable cycling beyond 1000 h, and LSB initial discharge capacity at 0.1C rises from 999 to 1201 mAh g^−1^. This universal grafting route can incorporate organosulfur active sites into diverse mesoporous MOFs, with promising extensions to metal anode protection, potassium–sulfur batteries, and general electrocatalysis.

## Abstract 2

Mechanism of action of D-MIL-101(Cr).

## Graphical abstract

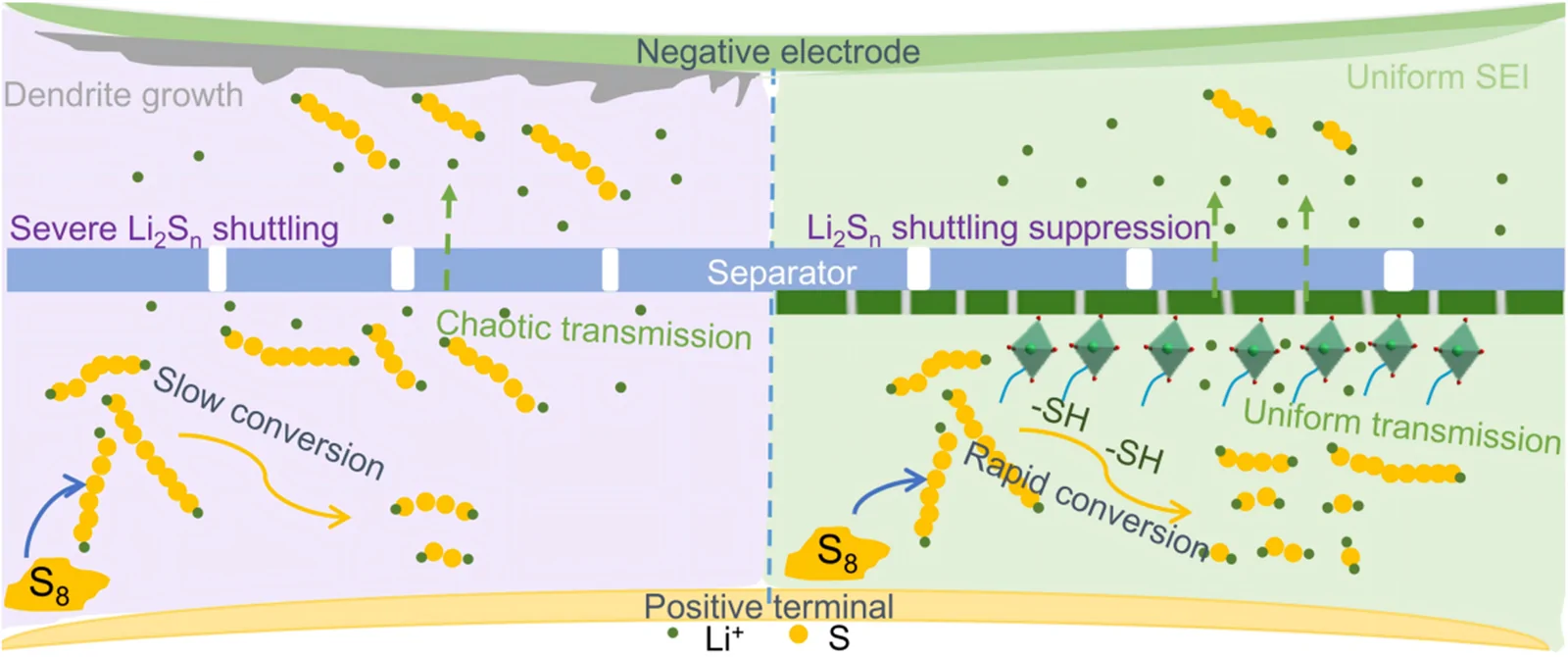

## 1. Introduction

Rising global energy demands have spurred advances in low-cost energy storage technologies that surpass the energy limits of conventional lithium-ion batteries (LIBs).^1–5^ Lithium–sulfur batteries, featuring high theoretical energy density and low raw material costs, stand out as leading next-generation storage candidates. Nevertheless, their practical energy output falls far below theoretical values, primarily due to soluble polysulfide intermediates. These dissolved species freely diffuse through porous separators toward lithium anodes, precipitating insoluble Li_2_S/Li_2_S_2_ that erode active sulfur and passivate metal lithium surfaces.^6,7^ Uneven Li^+^ transport additionally induces lithium dendrite growth, which raises risks of battery short-circuit. Accordingly, functional separators have been widely explored to boost sulfur utilization and extend LSB cycle lifespans.^8^

Functional separators represent an efficient strategy to mitigate polysulfide shuttling and boost lithium–sulfur battery performance.^9–11^ Unlike cathode or electrolyte modification, separators act as direct functional barriers to capture and convert diffusive polysulfides (LiPS) by blocking their transport channels.^12,13^ Conventional commercial separators are fabricated from polyolefins (polypropylene (PP) and polyethylene (PE)), yet they suffer intrinsic flaws: weak electrolyte affinity, poor thermal stability, and negligible capacity to restrain polysulfide crossover.^14^ Researchers have reported diverse coating materials—carbonaceous substances, metal oxides, sulfides and phosphides—to optimize separator functionality, targeting simultaneous suppression of shuttling and lithium dendrite proliferation.^15–22^ Unfortunately, most coatings depend merely on physical confinement, which incompletely blocks polysulfide permeation and barely alleviates dendrite growth.^23–25^ Balancing reliable battery safety and rapid ion transport remains difficult, creating an urgent demand for advanced separator structural engineering.^26–29^

Metal–organic frameworks (MOFs) are porous organic–inorganic crystalline hybrids with large specific surface areas, abundant pores, and structural versatility, rendering them promising candidates for catalysis and energy storage applications.^30^ Their tunable architectures allow deployment as electrodes, separators, and solid-state electrolytes in energy devices.^31–33^ Benefiting from confined pore spaces and plentiful open metal sites, MOFs facilitate fast Li^+^ transport and have been widely explored as separator coatings, with ZIF and MIL frameworks dominating related reports.^34–50^ Ordered MOF channels physically trap polysulfides, while unsaturated metal centers weakly coordinate these intermediates to partially mitigate shuttling and accelerate Li^+^ kinetics. Nevertheless, traditional metal–organic framework (MOF) separators still have significant drawbacks. They primarily rely on passive physical barriers to hinder polysulfide migration; their catalytic function is still largely determined by intrinsic or doped inorganic metal centers, which limits the conversion of polysulfides; and the vast majority are unable to ensure uniform Li^+^ deposition to suppress the growth of lithium dendrites.

Motivated by these drawbacks, we synthesize D-MIL-101(Cr) by covalently immobilizing dithiothreitol (DTT) onto the open metal sites of MIL-101(Cr) pores, then coat this composite onto PP separators for Li–S batteries. The DTT and MOF matrix jointly adsorb polysulfides, while free thiol (–SH) moieties streamline polysulfide redox pathways. Abundant lithiophilic –SH and –OH groups grafted on the framework, paired with size exclusion from ordered MOF channels, homogenize Li^+^ flux and promote steady lithium nucleation at the anode. It delivers robust polysulfide adsorption/catalysis on the cathode side and uniform Li^+^ migration on the anode side. Benefiting from dual cathode–anode interfacial modulation, the redox-active coating supports stable cycling of Li‖Li symmetric batteries beyond 1000 h. Li–S batteries assembled with this separator achieve drastically enhanced capacity and cyclability, raising the 0.1C initial discharge capacity from 999 to 1201 mAh g^−1^ and retaining steady performance over 500 cycles at 0.5C with an ultralow capacity decay of 0.077% per cycle. These results validate the great practical promise of redox-functionalized MOF separators with dual-interface regulatory capability.

## 2. Results and discussion

**Fig. 1:**
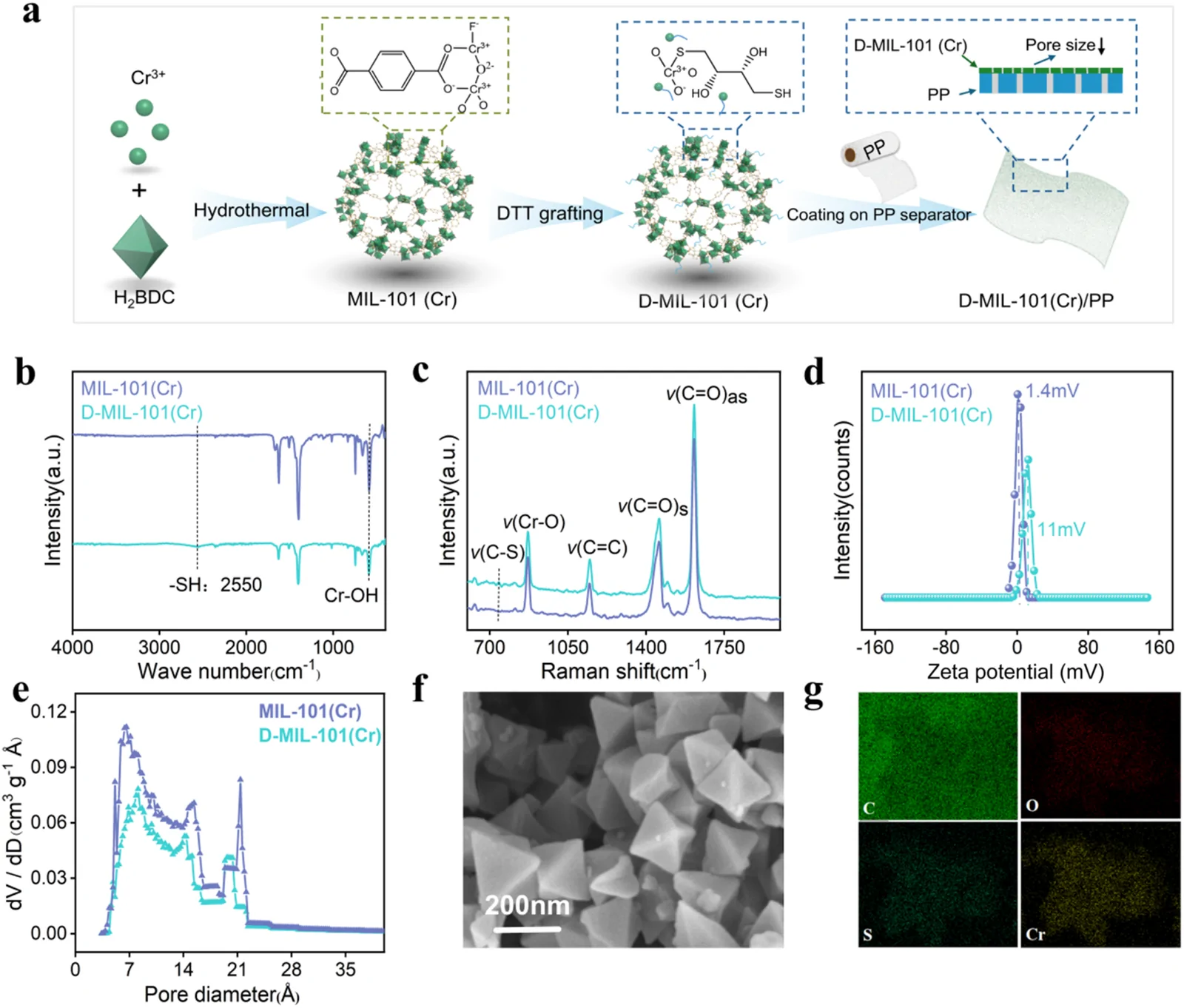
Synthesis and characterization of MOFs. (a) Schematic diagram of the synthesis and coating of D-MIL-101(Cr); (b) FT-IR spectra, (c) Raman spectra, (d) zeta potential plots and (e) pore size distribution plots of MIL-101(Cr) and D-MIL-101(Cr); (f) SEM image of D-MIL-101(Cr); (g) EDS elemental distribution map of D-MIL-101(Cr).

D-MIL-101(Cr) is synthesized *via* grafting DTT onto MIL-101(Cr) precursors. MIL-101(Cr) is chosen for its robust framework, matched pore dimensions and accessible open metal sites (OMSs) for guest immobilization, while DTT acts as a redox mediator capable of cleaving disulfide linkages in polysulfides. Its dual terminal –SH groups and 5.3 Å molecular size allow favorable chemical integration within MIL-101(Cr) pores (Fig. 1a). Herein, DTT molecules are anchored to the exposed trinuclear Cr_3_O octahedral OMSs of MIL-101(Cr); the resulting D-MIL-101(Cr) is coated onto standard PP separators for Li–S battery testing.

Raman and Fourier-transform infrared (FT-IR) spectroscopy were first employed to characterize DTT coordination within D-MIL-101(Cr). The FT-IR spectrum (Fig. 1b) displays a distinct *ν*(–SH) band at 2550 cm^−1^ exclusive to D-MIL-101(Cr), alongside a redshifted Cr–OH asymmetric stretching signal, confirming coordination between DTT sulfur atoms and framework Cr^3+^ centers to rearrange local metal coordination environments. Raman spectra (Fig. 1c) further feature a new C–S bending vibration at 740 cm^−1^, verifying DTT incorporation into the MOF lattice. Collectively, these spectroscopic data prove DTT anchors to MIL-101(Cr) *via* S–Cr coordination, embedding stable polar thiol sites inside pores to strengthen chemical trapping of lithium polysulfides through polar affinity. Zeta potential measurements (Fig. 1d) offer direct corroboration of successful DTT grafting. Pristine MIL-101(Cr) exhibits a zeta potential of 1.4 mV, while D-MIL-101(Cr) rises sharply to 11 mV. This positive shift originates from surface enrichment of thiol functional groups, which modify the MOF's surface chemical composition and charge density. The elevated surface potential electrostatically attracts negatively charged polysulfide anions, endowing the modified separator with intrinsic capacity to mitigate polysulfide shuttling. BET analysis (Fig. 1e) reveals reduced pore diameters after DTT functionalization. SEM (Fig. 1f and S1) and powder XRD (Fig. S2) show the ∼300 nm octahedral MIL-101(Cr) particles retain their complete morphological and structural integrity after grafting. Minor shifts and intensity variations in XRD peaks arise from guest DTT molecules occupying internal cavities and reorienting atomic arrangements on crystal planes. Energy-dispersive X-ray spectroscopy (EDS; Fig. 1g and Table S1) provided two lines of evidence confirming that DTT had been successfully immobilized within the MIL-101(Cr) scaffold. The sulfur imaging in Fig. 1g shows a uniform, evenly distributed S signal across the entire grain, which preliminarily rules out the possibility that DTT is merely physically adsorbed on the outer surface of the MOF particles and indicates that the thiol-containing molecules have penetrated into the internal mesoporous cavities, coming into contact with unsaturated Cr metal sites. The full-spectrum atomic percentage data summarized in Table S1 further support the semi-quantitative estimation of the relative S/Cr molar ratio: the measured sulfur atomic fraction was 0.68 at%, and the chromium atomic fraction was 1.64 at%. Given that each DTT molecule contains two sulfur atoms, we roughly calculated that each chromium center binds approximately 0.207 DTT molecules. Since the repeating structural unit of MIL-101(Cr) contains three chromium nodes, it follows that approximately 0.62 moles of DTT are grafted onto each mole of MIL-101(Cr) backbone unit. These results confirm that DTT has been successfully embedded within the mesoporous structure of MIL-101(Cr) without completely blocking the pores.

**Fig. 2:**
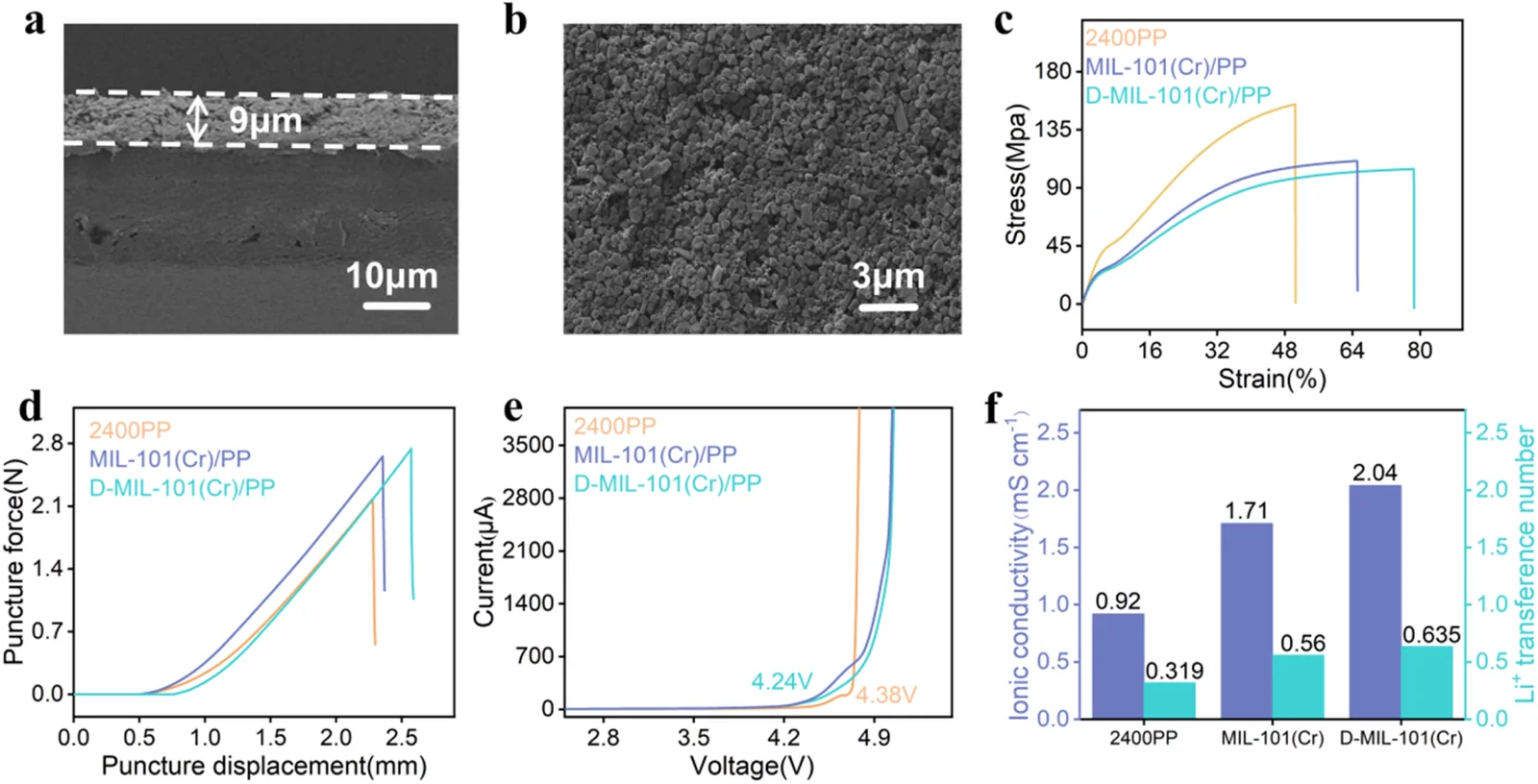
Basic properties of the separator. (a) SEM image of the cross-section of the D-MIL-101(Cr)/PP separator. (b) SEM image of the surface of the D-MIL-101(Cr)/PP separator. (c) Tensile stress–strain curves, (d) puncture curves, (e) electrochemical window curves, and (f) ion mobility and ionic conductivity of different separators.

Separator morphology and physical properties dominate polysulfide trapping, catalytic conversion and Li^+^ migration, so SEM was first applied to characterize bare PP and D-MIL-101(Cr)/PP separators (Fig. S3). Cross-sectional SEM (Fig. 2a) reveals the uniform D-MIL-101(Cr) coating layer has a uniform thickness of approximately 9 µm. All separators used for cell tests are cut into circular discs with a diameter of 19 mm. Each blank 19 mm PP separator weighs 4 mg, and the dry coating mass loaded on every single separator disc is 0.4 mg, corresponding to a mild mass increment of 10% and a coating mass fraction of ∼9% in the composite separator. Such a small additional mass will not severely sacrifice the gravimetric energy density of Li–S full cells. Bare PP possesses large macropores up to hundreds of nanometers, which permit free penetration of dissolved polysulfides. In comparison, Fig. 2b demonstrates the PP surface is fully covered by compact D-MIL-101(Cr) layers with uniform intrinsic micropores, which simultaneously block polysulfide crossover and retard lithium dendrite growth. The composite maintains its intact shape after repeated twisting (Fig. S4), confirming tight adhesion between the MOF layer and PP substrate with balanced mechanical rigidity and flexibility. Thermal dimensional stability is critical for battery safety. At 150 °C, the transverse thermal shrinkage of D-MIL-101(Cr)/PP falls sharply from 25% (pristine PP) to only 11% (Fig. S5). Such outstanding thermal resistance effectively avoids internal short circuits during high-temperature cycling. Contact angle and electrolyte uptake tests (Fig. S6 and S7) further verify the coating greatly enhances separator wettability: the contact angle approaches 0°, and electrolyte uptake reaches 77%. This benefit arises from the synergy between MOF porous frameworks and DTT-derived polar moieties, which offer abundant electrolyte reservoirs and continuous Li^+^ pathways to alleviate polysulfide shuttling and accelerate ion transport.

Mechanical strength and puncture tolerance are vital metrics for guaranteeing safe lithium–sulfur battery operation. Stress–strain and puncture tests (Fig. 2c and d) compare the mechanical properties of bare PP and D-MIL-101(Cr)/PP separators. Pristine PP delivers high tensile strength yet only ∼50% elongation at break. While D-MIL-101(Cr)/PP slightly sacrifices tensile strength, its elongation rises to ∼80%, reflecting markedly improved toughness. The composite also withstands larger maximum puncture force and displacement than bare PP, demonstrating enhanced tolerance against lithium dendrite piercing. Uniformly dispersed D-MIL-101(Cr) particles synergize with the PP substrate to boost deformability while retaining baseline tensile properties. Such robust mechanical characteristics greatly reduce the risk of battery short circuits, supporting stable long-term cycling. Linear sweep voltammetry (LSV, Fig. 2e) evaluates the electrochemical stability window of each separator. The bare 2400 PP separator oxidizes at 4.38 V, whereas D-MIL-101(Cr)/PP exhibits a marginally lower oxidation onset at 4.24 V. This minor decline originates from strong electrolyte affinity of the MOF's polar sites. Even so, the modified separator maintains a sufficiently wide stable voltage range compatible with standard Li–S battery cycling, with no premature severe oxidative decomposition observed.

Li‖Li and stainless-steel symmetric batteries (Fig. S8, S9 and 2f) were assembled to measure Li^+^ transference numbers and ionic conductivity, quantifying the separator's capacity to homogenize lithium-ion transport. The ionic transport performance of D-MIL-101(Cr)/PP was superior to that of bare PP and unmodified MIL-101(Cr)/PP. Bare 2400 PP provided an ionic conductivity of only 0.92 mS cm^−1^ and a Li^+^ migration number of 0.31; unmodified MIL-101(Cr)/PP reaches 1.71 mS cm^−1^ and 0.56; whereas D-MIL-101(Cr)/PP further improves these values to 2.04 mS cm^−1^ and 0.64. The increase in the lithium-ion mobility can be attributed to the grafted DTT molecules, which introduce a large number of highly electronegative –SH and –OH polar sites into the MOF mesopores. These sites form reversible, weak coordination bonds with Li^+^ cations and act as hopping sites to facilitate Li^+^ transport. At the same time, the bulky TFSI^−^ anions are severely restricted by the narrowing of the pore channels and the electrostatic repulsion between the S/O atoms on DTT, which significantly inhibits anion migration through the membrane. The increased selectivity for Li^+^ over anions raises the proportion of charge carried by lithium ions, leading to an increase in the *t*_Li^+^_ value. The improvement in electrical conductivity can be attributed to several synergistic effects. These include the significantly enhanced electrolyte wettability and adsorption rate of D-MIL-101(Cr), the lower activation energy for Li^+^ migration, and the fully interconnected mesoporous channels of the grafted MIL-101(Cr). The higher Li^+^ mobility, combined with faster ionic kinetics, collectively contribute to the enhanced ionic conductivity following DTT functionalization.

**Fig. 3:**
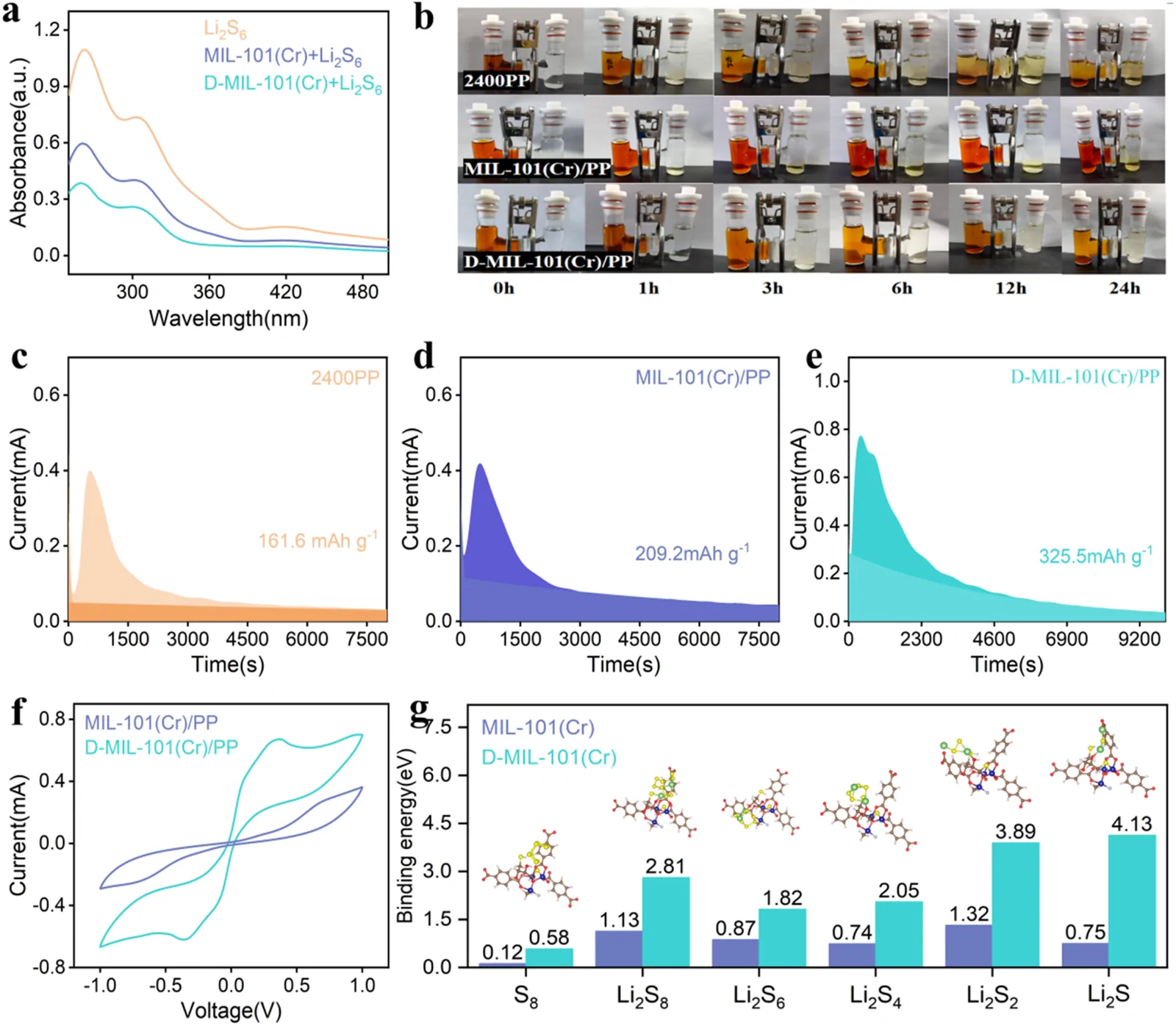
Adsorption and catalytic conversion of polysulfides. (a) UV-visible spectra of Li_2_S_6_ solution before and after the addition of metal–organic framework materials; (b) permeation experiment results in an H-type battery using different separators; (c–e) sulfur deposition experiments using different separators; (f) cyclic voltammetry curves of symmetric batteries with MIL-101(Cr)/PP and D-MIL-101(Cr)/PP electrodes in Li_2_S_6_-containing electrolyte at −1 to 1 V; (g) adsorption capacities of MIL-101(Cr) and D-MIL-101(Cr) for different polysulfides.

Efficient polysulfide adsorption and catalytic transformation are core requirements for advanced Li–S separators. Polysulfide static adsorption (Fig. 3a) and H-battery permeation measurements (Fig. 3b) verify that D-MIL-101(Cr)/PP delivers far stronger physical blocking and chemical anchoring toward polysulfides than bare PP and pristine MIL-101(Cr)/PP. UV-vis spectra of Li_2_S_6_ solutions reveal drastically weakened characteristic absorption bands after introducing MOF samples, with the most dramatic signal decay observed for D-MIL-101(Cr), proving its superior polysulfide affinity. After 24 h of H-battery testing, the compartment separated by bare PP turns deep yellow from unobstructed polysulfide crossover; MIL-101(Cr)/PP offers limited mitigation yet still allows obvious color leakage, whereas only faint yellow emerges across the D-MIL-101(Cr)/PP barrier. This outstanding suppression arises from dual synergism: MOF pores physically intercept soluble intermediates, and surface polar sites chemically immobilize polysulfide species. Collectively, these adsorption tests validate that DTT grafting greatly boosts MIL-101(Cr)'s polysulfide-trapping capacity.

Constant-potential Li_2_S deposition measurements (Fig. 3c–e) quantitatively assess each separator's catalytic ability to drive liquid polysulfide conversion into solid Li_2_S_2_/Li_2_S. Bare 2400 PP delivers faint reduction currents and merely 161.6 mAh g^−1^ of Li_2_S deposition capacity, reflecting negligible catalytic activity that fails to convert soluble long-chain polysulfides and exacerbates shuttling. Unmodified MIL-101(Cr)/PP slightly improves deposition capacity to 209.2 mAh g^−1^*via* intrinsic unsaturated metal centers, yet its catalytic performance remains constrained. By comparison, D-MIL-101(Cr)/PP displays the sharpest initial reduction peak and longest reaction duration, achieving a boosted deposition capacity of 325.5 mAh g^−1^. The grafted redox thiol moieties cooperate with MIL-101(Cr) metal sites to construct a synergistic catalytic network that reduces the energy barrier for liquid–solid polysulfide transformation. This composite rapidly sequesters free long-chain intermediates and facilitates their full precipitation, fundamentally restraining polysulfide buildup and mitigating the shuttle effect. Cyclic voltammetry of polysulfide symmetric batteries (Fig. 3f) further corroborates that D-MIL-101(Cr) accelerates polysulfide redox kinetics, consistent with the sulfur deposition outcomes.

Density functional theory (DFT) calculations (Fig. 3g and S10) reveal stronger adsorption and catalytic activity of D-MIL-101(Cr) toward diverse sulfur species relative to pristine MIL-101(Cr). This improvement originates from dual intermolecular interactions between DTT's thiol moieties and lithium polysulfide fragments. Meanwhile, D-MIL-101(Cr) possesses much lower free-energy barriers for long-to-short polysulfide conversion, verifying that redox-active DTT functionalities substantially upgrade the MOF's polysulfide regulation performance. Fig. S11 and S12 depict the plausible reaction mechanism. On the separator's cathode face, D-MIL-101(Cr) captures lithium polysulfides, whose sulfur atoms coordinate with the framework's exposed thiol groups. Progressive discharge initiates successive homolytic bond cleavage to yield sulfur radicals and Li^+^, finally producing D-MIL-101(Cr)–Li and solid Li_2_S. Unlike the multi-step redox pathway of batteries with PP separator, the modified MOF mediates rapid interconversion of short organosulfur intermediates, shortening the overall sulfur redox cascade. Collectively, DFT simulations clarify the optimized sulfur reaction routes and underline the theoretical superiority of D-MIL-101(Cr) for lithium–sulfur battery systems.

**Fig. 4:**
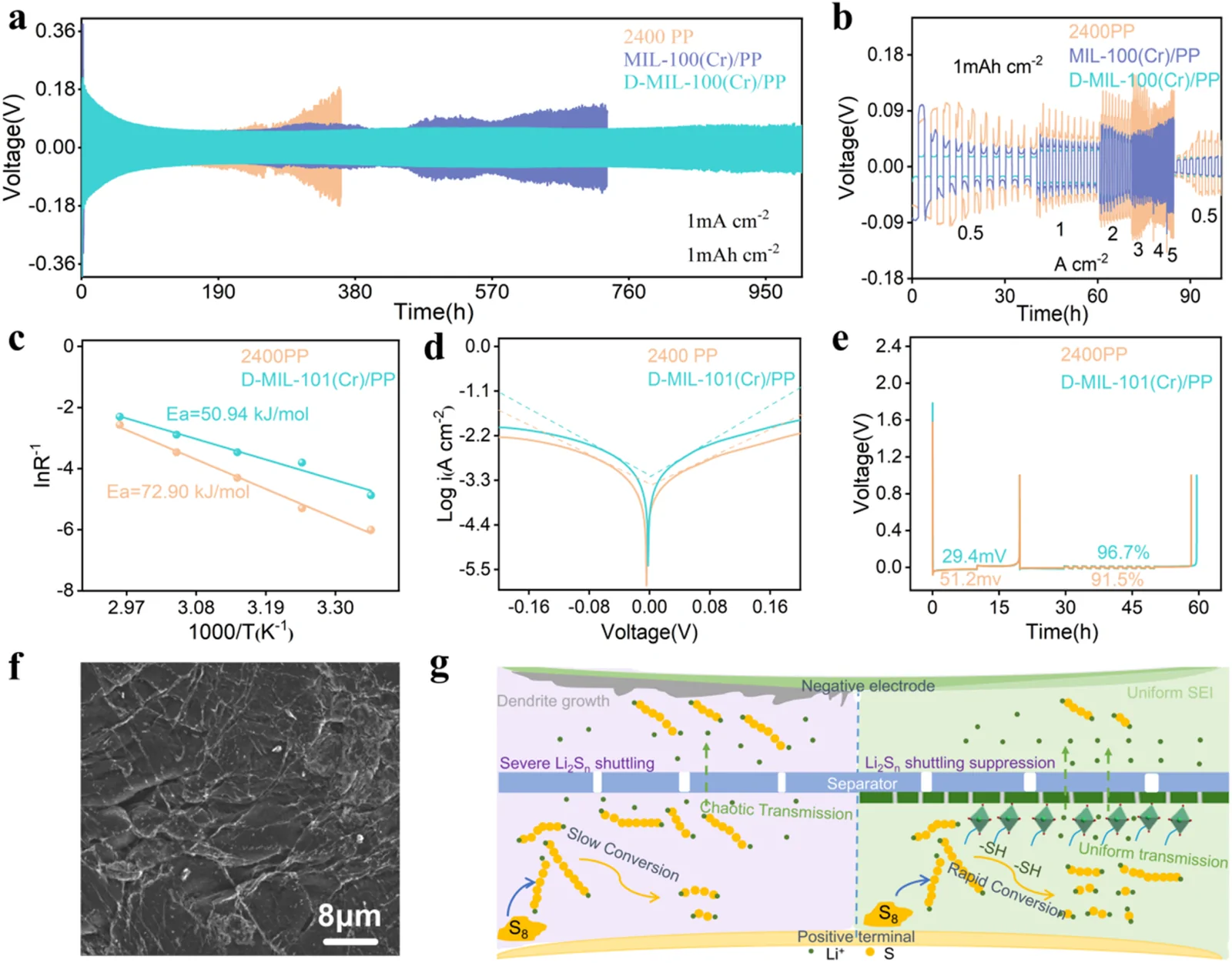
Uniform lithium-ion transport. (a) Long-cycle performance of Li‖Li batteries with different separators. (b) Lithium deposition/stripping performance at different current densities in Li‖Li batteries using different separators. (c) Reaction activation energies of lithium–lithium symmetric batteries using 2400 PP and D-MIL-101(Cr)/PP (*E*_a_). (d) Tafel curves for Li‖Li batteries with 2400 PP and D-MIL-101(Cr)/PP. (e) Average coulombic efficiency of Li‖Cu batteries with 2400 PP and D-MIL-101(Cr)/PP. (f) Surface morphology of lithium metal after cycling in Li‖Li batteries with D-MIL-101(Cr)/PP. (g) Schematic diagram of the functionality of D-MIL-101(Cr)/PP.

Lithium dendrite proliferation severely degrades battery performance, so ideal separators must simultaneously homogenize Li^+^ flux and restrain dendrite nucleation. Electrochemical characterizations were carried out to validate the regulatory effect of D-MIL-101(Cr)/PP on lithium transport. Cyclic voltammetry of Li‖Li batteries (Fig. S13) delivers stronger redox peak currents for D-MIL-101(Cr)/PP than bare 2400 PP, confirming accelerated lithium deposition/stripping kinetics. Long-term galvanostatic cycling (Fig. 4a and S14) further evaluates anode interfacial stability. Batteries assembled with bare PP suffer steadily rising polarization and severe voltage noise originating from chaotic dendrite growth and enlarged interfacial resistance. In contrast, D-MIL-101(Cr)/PP maintains steady voltage profiles with minimal polarization over 1000 h, reflecting highly reversible lithium plating/stripping. Rate measurements (Fig. 4b) show drastic polarization surging and collapsed voltage plateaus for bare PP as current rises from 0.5 to 5 mA cm^−2^. Unmodified MIL-101(Cr)/PP partially alleviates this issue yet retains obvious voltage hysteresis at high rates. By comparison, D-MIL-101(Cr)/PP sustains the lowest polarization across all current densities and quickly recovers stability after rate switching, showcasing outstanding rate tolerance. Such advantages stem from the D-MIL-101(Cr) coating: it optimizes electrolyte infiltration and continuous ion channels, while homogenized Li^+^ flux inhibits irregular dendrite formation and stabilizes the lithium anode interface.

Variable-temperature EIS, Arrhenius activation energy and Tafel measurements systematically unravel how D-MIL-101(Cr)/PP modulates Li^+^ migration kinetics and anode interfacial energy barriers in Li–S batteries. EIS spectra (Fig. S15) demonstrate falling impedance for all separators upon heating from 25 to 65 °C. Nevertheless, D-MIL-101(Cr)/PP consistently delivers far lower impedance with more dramatic semicircle shrinkage under heating, signifying thermally responsive, faster ion transport. Arrhenius fitting (Fig. 4c) quantifies this gap: the modified separator yields an ion migration activation energy of 50.94 kJ mol^−1^, much smaller than the 72.90 kJ mol^−1^ recorded for bare PP. This decline arises from synergy between DTT-derived polar moieties and MOF porosity, which collectively lower Li^+^ conduction barriers and construct uninterrupted ion pathways. Tafel profiles (Fig. 4d) align perfectly with activation energy trends: D-MIL-101(Cr)/PP features elevated exchange current density and gentler polarization slopes, representing expedited lithium plating/stripping with milder interfacial resistance. Collectively, the coating cuts Li^+^ migration barriers and accelerates interfacial redox kinetics, laying a solid foundation for prolonged stable battery cycling.

The dual regulation of lithium-ion migration and lithium deposition stems from the synergistic interaction between the intrinsic mesoporous sieving effect of the MOF and the D-derived lithium-affinity sites, which can be elucidated layer by layer through electrochemical characterization. BET pore size analysis shows that the average pore size after DTT grafting is approximately 11 Å. This size matches the hydrodynamic size of solvated lithium ions and simultaneously allows for the physical sieving of larger, long-chain polysulfide anions, thereby enabling smooth lithium-ion transport while preventing anode side reactions caused by shuttling. Although the pore size contracted slightly after DTT immobilization, the interconnected mesoporous network was fully preserved, providing uniform ionic pathways within the membrane plane. At the same time, a series of characterizations collectively confirmed that DTT modification restructured the lithium-ion coordination environment within the MOF pores. The dense concentration of electronegative –SH/–OH groups on the inner walls of the channels causes the zeta potential to rise significantly from 1.4 mV to 11 mV, and DFT adsorption energy calculations also demonstrate a substantial enhancement in the binding between the sulfur intermediates and D-MIL-101(Cr); the uniformly distributed S/O sites can form reversible, uniform coordination with solvated Li^+^, compensating for the sparse and weak Li^+^–Cr–O interactions in the original MIL-101(Cr) and eliminating localized high-energy nucleation hotspots that induce non-uniform lithium growth. We further distinguished the primary and secondary factors influencing stable lithium deposition using quantitative electrochemical metrics. The lithium ion migration number increased from 0.56 in the original MIL-101(Cr) to 0.64 after grafting, which suppresses anion accumulation on the lithium anode surface and alleviates severe concentration polarization, a key factor in inhibiting dendrite formation. Meanwhile, the reduction in interfacial charge transfer impedance and the increase in Tafel exchange current density serve only as auxiliary conditions, accelerating the uniform lithium redox kinetics. These patterns are intuitively corroborated by the Li‖Cu tests in Fig. 4e and f, as well as the morphology of the lithium anode after cycling. Li‖Cu battery measurements (Fig. 4e) verify D-MIL-101(Cr)/PP greatly enhances the reversibility of lithium plating and stripping. The modified battery delivers an average polarization of merely 29.4 mV and an average coulombic efficiency of 96.7%, outperforming bare PP (51.2 mV, 91.5%). This contrast confirms the coating lowers interfacial barriers to drive homogeneous Li deposition. Post-cycling SEM of Li‖Li anodes (Fig. 4f and S16) offers direct morphological evidence for dendrite inhibition. The bare PP paired anode appears rough, cracked and covered with stacked dendrites, whereas D-MIL-101(Cr)/PP yields smooth, dendrite-free lithium surfaces. The grafted MOF provides continuous ion pathways and abundant lithiophilic sites to equalize Li^+^ flux and stabilize anode interfaces.

Fig. 4g illustrates the dual-functional mechanism of D-MIL-101(Cr)/PP separator. On the anode-facing side of the separator, the intrinsic pores of MIL-101(Cr) and lithiophilic polar –SH/–OH groups from DTT serve as uniform Li^+^ transport channels, which eliminate local Li^+^ concentration gradients and effectively restrain irregular lithium dendrite nucleation and growth. On the cathode-facing side, the composite coating realizes dual polysulfide restriction effects *via* physical pore sieving and strong chemical polar anchoring, while the synergistic Cr metal centers and redox-active thiol groups significantly lower the energy barrier for liquid-to-solid conversion of long-chain lithium polysulfides, fundamentally mitigating the polysulfide shuttle effect that severely plagues bare PP separators. The two sides of the separator perform independent yet complementary interfacial regulatory functions. At the same time, through a systematic structural and electrochemical comparison of MIL-101(Cr)/PP and D-MIL-101(Cr)/PP, we further distinguished the respective contributions of the MIL-101(Cr) inherent porous framework and the grafted redox-active DTT groups. In terms of the catalytic conversion of polysulfides, D-MIL-101(Cr) significantly enhances the Li_2_S deposition capacity compared to MIL-101(Cr)/PP. Furthermore, DFT calculations show that the sulfur–mercaptan redox interactions originating from the –SH groups accelerate the liquid–solid polysulfide conversion process. Regarding changes in the porous structure after grafting, BET characterization data indicate that the specific surface area decreased moderately from 2656 m^2^ g^−1^ (for the original MIL-101(Cr)) to 2041 m^2^ g^−1^, accompanied by slight pore shrinkage; however, the interconnected mesoporous channels remained intact, and this minor structural sacrifice was offset by the functional advantages of the polar thiol sites. In terms of lithium-ion transport, compared to MIL-101(Cr)/PP, D-MIL-101(Cr) exhibited a significant increase in the Li^+^ transfer number and a reduction in the activation energy for Li^+^ migration, which is primarily attributed to selective Li^+^ transfer sites and the anion-repulsion effect of the thiol groups. In summary, the MIL-101(Cr) scaffold provides a stable porous support structure and weak polysulfide adsorption capacity, while the grafted DTT introduces unique redox catalytic activity and lithium-affinity sites, which are the primary sources of performance enhancement; the synergy between the two enables dual-interface regulation of polysulfide transformation and homogeneous Li^+^ flux.

**Fig. 5:**
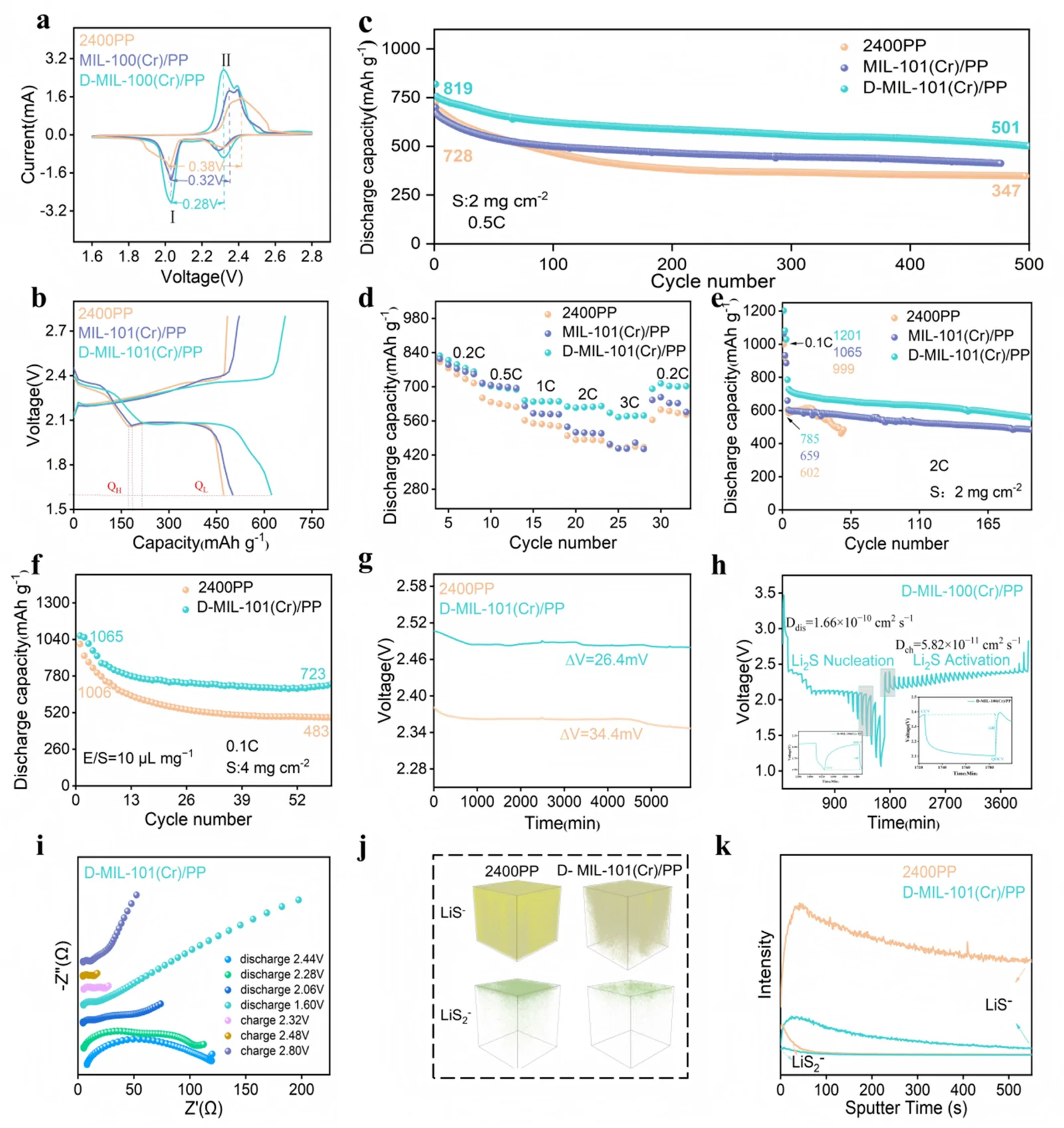
Performance testing of lithium–sulfur batteries. (a) Cyclic voltammetry curves for different separators; (b) constant-current charge–discharge curves of lithium–sulfur batteries with different separators at 0.5C; (c) long-cycle performance of lithium–sulfur batteries with different separators at 0.5C; (d) rate performance of lithium–sulfur batteries with 2400 PP and D-MIL-101(Cr)/PP separator; (e) long-cycle performance of lithium–sulfur batteries with different separators at 2C; (f) long-cycle performance of high-sulfur-loading lithium–sulfur batteries with 2400 PP and D-MIL-101(Cr)/PP separator at 0.1C; (g) changes in self-discharge voltage of batteries with 2400 PP and D-MIL-101(Cr)/PP separator; (h) GITT test of lithium–sulfur batteries with D-MIL-101(Cr)/PP separator; (i) *in situ* EIS of lithium–sulfur batteries with D-MIL-101(Cr)/PP separator; (j) time-of-flight secondary ion mass spectrometry (TOF-SIMS) analysis of LiS^−^ and LiS_2_^−^ content on the surface of the anode lithium foil after 15 cycles for batteries with 2400 PP and D-MIL-101(Cr)/PP separator; yellow represents LiS^−^, green represents LiS_2_^−^; (k) schematic illustration of LiS^−^ and LiS_2_^−^ content in the TOF-SIMS analysis.

Assembled Li–S full batteries were evaluated for comprehensive electrochemical performance. Cyclic voltammetry (Fig. 5a) displays two canonical redox pairs for sulfur species: the ∼2.0 V reduction peak (I) reflects sequential transformation of long-chain polysulfides into Li_2_S_4_ and ultimately solid Li_2_S_2_/Li_2_S, while the 2.3–2.4 V oxidation peak (II) corresponds to reverse reconversion into high-order polysulfides. Batteries equipped with D-MIL-101(Cr)/PP deliver markedly sharper, higher-current redox peaks than bare PP and pristine MIL-101(Cr)/PP, signifying expedited polysulfide redox kinetics. Galvanostatic profiles at 0.5C (Fig. 5b) further validate the coating's strong catalytic activity toward short-chain sulfide deposition. Long-cycle stability at 0.5C (sulfur loading: 2 mg cm^−2^, Fig. 5c) highlights the superiority of D-MIL-101(Cr)/PP. It delivers an initial discharge capacity of 819 mAh g^−1^ and maintains 501 mAh g^−1^ over 500 cycles, corresponding to an ultralow capacity fade of 0.077% per cycle. By contrast, bare PP only reaches an initial capacity of 728 mAh g^−1^ and fades drastically to 347 mAh g^−1^ at a decay rate of 0.11% per cycle; unmodified MIL-101(Cr)/PP exhibits intermediate performance. These cycling and voltage profile results collectively prove the abundant active sites on D-MIL-101(Cr) accelerate polysulfide interconversion, mitigate the shuttle effect, and boost sulfur utilization efficiency.

Rate capability measurements (Fig. 5d and S17) confirm D-MIL-101(Cr)/PP elevates Li–S battery rate performance and redox reversibility. Across stepped current densities from 0.2C to 3C, batteries with D-MIL-101(Cr)/PP deliver higher discharge capacities at every rate, reflecting superior rate tolerance. Corresponding galvanostatic profiles show flatter charge/discharge plateaus and reduced voltage polarization relative to bare PP; the modified separator also exhibits minimal plateau distortion under elevated currents. Robust polysulfide anchoring and catalytic conversion from the D-MIL-101(Cr) coating restrain shuttling, sustaining steady battery output under high-rate operation. Long-term cycling at 2C (sulfur loading 2 mg cm^−2^, Fig. 5e) further verifies the coating's stabilizing effect. Batteries with bare PP separator yield initial capacities of 999 mAh g^−1^ at 0.1C and 602 mAh g^−1^ at 2C, while unmodified MIL-101(Cr)/PP reaches 1065 and 659 mAh g^−1^ under identical conditions. In contrast, D-MIL-101(Cr)/PP delivers markedly improved discharge capacities of 1201 mAh g^−1^ at 0.1C (20% higher than bare PP) and 785 mAh g^−1^ at 2C (30% higher than bare PP), outperforming both control separators in this work, and retains substantially higher capacity over 200 cycles than MIL-101(Cr)/PP. Bare PP delivers the weakest cyclability of the three groups, underscoring the indispensable synergistic effect of the MOF scaffold and grafted DTT. Such outstanding high-rate durability originates from dual interfacial modulation, which simultaneously mitigates polysulfide crossover and lithium dendrite proliferation for durable Li–S cycling. It is worth noting that both the aforementioned cycle rates and long-cycle tests were conducted using a standard laboratory button cell system; detailed device parameters can be found in the SI. Although the current sulfur loading and test system have demonstrated excellent electrochemical stability, this laboratory button cell system still falls short of the thin-film design requirements for practical batteries. Further optimization of the practical battery system will bring this separator system closer to meeting the technical standards for practical lithium–sulfur energy storage devices.

Long-cycle galvanostatic measurements at a high sulfur loading of 4 mg cm^−2^ (Fig. 5f and S18) further confirm the performance boost afforded by D-MIL-101(Cr)/PP separators. At 0.1C, batteries with D-MIL-101(Cr)/PP deliver an initial discharge capacity of 1065 mAh g^−1^ and retain 723 mAh g^−1^ after 60 cycles. By contrast, bare PP starts at 1006 mAh g^−1^ and suffers severe capacity fading down to 483 mAh g^−1^ after 60 cycles. Galvanostatic profiles reveal flatter plateaus and diminished polarization for D-MIL-101(Cr)/PP, which preserves clear dual voltage plateaus even under high sulfur loading to support efficient, reversible polysulfide transformation. Batteries with bare PP separator display obvious plateau collapse and aggravated polarization in later cycles, triggered by severe polysulfide shuttling and massive active sulfur loss. Although cycling tests under more practical ultrahigh sulfur loading (>5 mg cm^−2^) are not included in this work due to current electrode fabrication limitations, the stable cycling behavior at 4 mg cm^−2^ already demonstrates its promising potential toward practical Li–S devices. Self-discharge evaluations (Fig. 5g and S19) highlight the coating's ability to mitigate spontaneous capacity loss. After 100 h of open-circuit rest, batteries with bare PP separator experience a 34.4 mV voltage drop, while D-MIL-101(Cr)/PP only decays by 26.4 mV, reflecting superior static voltage stability. Capacity retention tests further quantify this improvement: bare PP and D-MIL-101(Cr)/PP deliver initial capacities of 942 and 1143 mAh g^−1^, respectively; following 100 h storage, the battery with bare PP separator fades sharply to 723 mAh g^−1^, whereas the battery with modified separator sustains 948 mAh g^−1^. This discrepancy originates from synergistic polysulfide confinement by MOF pore channels and grafted thiol polar sites, which suppress shuttle-driven parasitic side reactions during shelf storage. Such outstanding anti-self-discharge capability improves the practical reliability of Li–S batteries equipped with this functional separator.

Galvanostatic intermittent titration technique (GITT) measurements (Fig. 5h and S20) confirm D-MIL-101(Cr)/PP accelerates polysulfide redox kinetics in Li–S batteries. The labeled charge–discharge profiles in Fig. 5h show the modified separator delivers elevated Li^+^ diffusion coefficients during both charge and discharge, which diminishes solid-state ion transport resistance and speeds up Li^+^ migration. Internal reaction resistance is quantified *via* the reported formula:^51–53^ Δ*R*_(internal)_ (Ω) = |Δ*V*_(QOCV–CCV)_|/*I*_(applied)_, where Δ*V* represents the voltage difference between the quasi-open-circuit voltage (QOCV) and closed-circuit voltage (CCV) points, and *I*_(applied)_ denotes the applied current. Batteries with D-MIL-101(Cr)/PP display smaller internal resistance at Li_2_S nucleation and activation stages, as the coating cuts thermodynamic reaction barriers and facilitates full sulfide conversion during cycling. *In situ* electrochemical impedance spectroscopy (EIS, Fig. 5i and S21) tracks dynamic interfacial impedance evolution of batteries with PP and D-MIL-101(Cr)/PP separator throughout charge–discharge cycles. Though impedance varies regularly for both groups with cycling, D-MIL-101(Cr)/PP maintains consistently lower impedance across all voltage windows. Such improvement originates from synergistic polysulfide chemical trapping and catalytic transformation, which inhibits intermediate buildup at electrode–electrolyte interfaces, lowers dynamic reaction impedance, and enables highly reversible sulfur redox.

Time-of-flight secondary ion mass spectrometry (ToF-SIMS) measurements (Fig. 5j and k) confirm the D-MIL-101(Cr)/PP modified separator effectively suppresses polysulfide shuttling in Li–S batteries. The spectrum (Fig. 5k) shows that LiS^−^ and LiS_2_^−^ signals on the lithium anode of the battery with bare PP separator are significantly higher and remain elevated even after prolonged sputtering, indicating massive polysulfide penetration and anode accumulation. In contrast, the battery with modified separator exhibits drastically reduced LiS^−^ and LiS_2_^−^ signals that decay rapidly throughout sputtering, verifying the coating's ability to block polysulfide migration. Consistent with the adsorption and catalytic conversion results in Fig. 3, these findings provide direct experimental evidence that the separator's dual-interface regulation mitigates the shuttle effect and enhances battery cycling stability.

Table 1 summarizes the core structures and electrochemical performance metrics of cutting-edge MOF separators in recent years, covering the substrate framework, functional groups, catalytic active sites, sulfur loading, liquid sulfur ratio, and long-cycle durability. The vast majority of existing MOF separators rely solely on inorganic metal nodes or static polar groups to achieve polysulfide adsorption and weak catalysis. Such structures generally have limited catalytic capacity, and most are unable to simultaneously and fully address the two core defects of lithium–sulfur batteries. In contrast, this modular post-grafting strategy immobilizes the redox-active DTT within the pores while fully retaining the crystalline pore structure of MIL-101(Cr); the grafted thiol groups act as dynamic redox mediators to accelerate the liquid-to-solid phase conversion of polysulfides, while a large number of lithium-affinity –SH/–OH sites simultaneously balance the lithium-ion flux and suppress dendrite growth. Thanks to this dual synergistic effect, the D-MIL-101(Cr) separator exhibits excellent cycling stability under both conventional operating conditions (2 mg cm^−2^, E/S = 20 µL mg^−2^) and high-loading, low-electrolyte conditions (4 mg cm^−2^, E/S = 10 µL mg^−2^). Compared to incremental improvements in the single functions of traditional MOF frameworks, this design provides a universal molecular engineering platform for the construction of multifunctional separators.

**Table 1:** Comparison of representative articles on MOF modification

Article	Basic MOFs	Introduction unit	Primary mechanism of action	Sulfur loading (mg cm^−2^)	E/S (µL mg^−2^)	Performance
Razaq *et al.*, *J. Power Sources*, 2024 (ref. 54)	UiO-66(Zr)-NH_2_	Introduction of Fe^3+^ into Zr-MOF nodes	Nanopore LiPS capture + Fe-site catalysis	2.7	10	1038 mAh g^−1^@0.1C
Kang *et al.*, *Nano-Micro Lett.*, 2025 (ref. 55)	Ti-MOF	–NH_2_ ligand functionalization	0.83 nm pore sieve + electrostatic adsorption + Lewis acid–base interaction	0.84–1.0	None	1079.8 mAh g^−1^ at 0.5C; 153 cycles at a high loading of approximately 3.0 mg cm^−2^
Liu *et al.*, *Adv. Sci.*, 2025 (ref. 56)	UiO-66	–NH_2_ + –SO_3_H bifunctional group	LiPS coordination/adsorption + Li^+^ coordination transport; multifunctional synergy	1.1–1.3	15	1215.7 mAh g^−1^@0.5C; 2C 500 laps, 533.4, approximately 0.085% per lap
Xu *et al.*, *Adv. Sci.*, 2025 (ref. 57)	Ni/Co-HHTP two-dimensional conjugated MOF	Ni/Co bimetallic redox node	d–π conjugated + Ni/Co redox-active metal sites catalyze LiPS	1.1–1.3	15	1148 mAh g^−1^ at 0.1C; 500 cycles at 1C, approximately 0.08% per cycle
Wang *et al.*, *Chem. Eng. J.*, 2025 (ref. 58)	UiO-66-NH_2_ + Ti_3_C_2_T_*x*_ MXene	MOF/MXene composite heterogeneous interfaces	MOF pores + MXene conductive/polar interface	1	35	1247 mAh g^−1^@0.1C
Zhao *et al.*, *Nano-Micro Lett.*, 2026 (ref. 59)	AHF-DPDC	Fe: monometallic anchoring; Zn: regulation of electronic structure	1D charged-channel screening + Fe-centered electrocatalytic LiPS	3.12	16	4.89 mg cm^−2^, E/S = 8: 791.9 → 654.1@0.5C/50 revolutions
Razaq *et al.*, *Adv. Energy Mater.*, 2024 (ref. 60)	ZIF-8	Fe doping → Fe-ZIF-8	Pore-screen + Fe metal active site electrocatalytic LiPS	1	None	863 mAh g^−1^@0.5C, 746@3C
This work, D-MIL-101(Cr)/PP	MIL-101(Cr)	DTT post-grafting; –SH/–OH; organic sulfur redox sites	Pore sieve + LiPS adsorption + thiol-mediated conversion + uniform Li^+^/Li deposition at lithium-accepting sites	2; high load 4.0	20; high load 10	1201 mAh g^−1^@0.1C; 0.5C, 500 cycles: 819 → 501, 0.077% per cycle; 4 mg cm^−2^: 1065; Li‖Li > 1000 h

## 3. Conclusion

This work reports a PP separator functionalized with DTT-grafted MIL-101(Cr) for dual cathode–anode interfacial modulation. On the cathode side, the coating traps polysulfides and accelerates their redox conversion; on the anode side, it homogenizes Li^+^ flux. DTT-derived polar moieties greatly boost the MOF's polysulfide anchoring and catalytic capacity, outperforming pristine MIL-101(Cr). The battery with modified separator alleviates self-discharge and enables Li‖Li symmetric batteries to sustain stable cycling over 1000 h. Li–S full batteries with D-MIL-101(Cr)/PP separator retain steady performance for 500 cycles at 0.5C, deliver an initial capacity of 785 mAh g^−1^ at 2C, and maintain favorable capacity retention and voltage profiles under a high sulfur loading of 4 mg cm^−2^. Benefiting from synergy between the MOF scaffold and thiol (–SH) groups, the separator concurrently restrains polysulfide shuttling and guides uniform lithium deposition.

This study offers a facile strategy to construct high-performance Li–S separators. This DTT post-grafting protocol represents a generalizable modular molecular engineering strategy decoupling MOF framework synthesis from pore functionalization, not limited to MIL-101(Cr). Enabled by coordinative binding to universal open metal sites, analogous thiol molecules can deliver comparable polysulfide-regulating functions despite variable activity dependent on thiol number and molecular geometry. Distinct from pre-functionalized ligand synthesis that alters intrinsic pore structures, this mild post-modification fully retains parent MOF topology. Rationally transferable to K–S batteries, metal anode protection and sulfur electrocatalysis *via* shared thiol–alkali metal coordination, the cross-system generality awaits further experimental validation on diverse MOF platforms in follow-up work.

## Author contributions

Yu Li conceptualized the study and contributed to the design and drafting of the manuscript. Kaiyan Wu, Cheng Zhang, Zhuangzhuang Zhang, Bofang Shi, Chen Liu, Suyue Guo and Zhicheng Zhang participated in the experimental work and the revision of the article. All authors reviewed and approved the final version of the manuscript. Mingbo Ma and Honghui Yang served as corresponding author and ensured the overall quality and completion of the work.

## Conflicts of interest

The authors declare no conflict of interest.

## Supplementary Material

SC-OLF-D6SC05954F-s001

## Data Availability

The data that support the findings of this study are available on request from the corresponding author upon reasonable request. Supplementary information (SI) is available. See DOI: https://doi.org/10.1039/d6sc05954f.
