# Supplementary material for: Grafting redox-active thiols into MOF channels for dual-interfacial regulation in Li–S batteries
Source: Chem Sci. 2026 Aug 28;17(40):19269–82. doi: 10.1039/d6sc05954f (PMC13522951; doi:10.1039/d6sc05954f)
Supplement: Supplementary file 1 [file SC-017-D6SC05954F-s001.pdf]

Supplementary Information (SI) for Chemical Science.
This journal is © The Royal Society of Chemistry 2026

Electronic Supplementary Information

Experimental Procedures

Materials

Chromium(III) nitrate nonahydrate ($CrNO_3 \cdot 9H_2O$, 99.95%, Aladdin), DL-Dithiothreitol (DTT, 99%, Zancheng (Tianjin) Technology Co., Ltd.), Terephthalic acid (H_2BDC, 99%, Aladdin), Conductive carbon black (Anhui Zesheng Technology Co., Ltd.), Hydrofluoric acid (40%, Guangzhou Chemical Reagent Factory), N, N-dimethylformamide (DMF, 99.9%, Shanghai Aladdin Biotechnology Co., Ltd.), chloroform (Shanghai Aladdin Biotechnology Co., Ltd.), polyvinylidene fluoride (PVDF, Shanghai Aladdin Biotechnology Co., Ltd.), sublimed sulfur (99.5%, Shanghai Aladdin Biotechnology Co., Ltd.), N-methylpyrrolidone (NMP, 99.5%, Beijing Kaiguo Technology Co., Ltd.), methanol (99.5%, Shanghai Aladdin Biotechnology Co., Ltd.), S/C composite material (S content 75%, Dongguan Kelude Laboratory Equipment Technology Co., Ltd.), lithium sulfide (Li_2S, 99%, Shanghai Aladdin Biochemical Technology Co., Ltd.). All chemical reagents were used directly without further processing.

Preparation of MIL-101(Cr)

Dissolve $CrNO_3 \cdot 9H_2O$ (800 mg, 2 mM) and terephthalic acid (332 mg, 2 mM) in 14 mL of deionized water, and add 100 μL of hydrofluoric acid. Transfer the solution to a PTFE-lined autoclave and sonicate for 10 minutes. The autoclave was then placed in an oven at 220°C for 8 hours. After cooling to room temperature, the precipitate was thoroughly washed with DMF and methanol, respectively.

Preparation of D-MIL-101(Cr)

First, 0.496 g of DTT was dissolved in 10 mL of chloroform to form a uniform, transparent solution under an argon atmosphere. Next, 200 mg of MIL-101(Cr) powder was activated for 12 hours under vacuum at 150°C to remove the coordinated water from the MOF. Before the MOF powder cooled, the DTT solution was rapidly added. The mixture was then stirred overnight, and the D-MIL-101(Cr) sample was collected by filtration and washed several times with chloroform.

Preparation of the S Cathode

The S/C composite was uniformly mixed with conductive carbon black and PVDF in a mass ratio of 8:1:1. The mixture was stirred in a solution of N-methylpyrrolidone and coated onto carbon-coated aluminum foil using a 300-μm squeegee. It was then vacuum-dried at 60°C for 12 hours. The loading was 2 mg/cm^2.

Preparation of Modified Separators

The test samples (MIL-101(Cr) and D-MIL-101(Cr)) were mixed with polyvinylidene fluoride (PVDF) and conductive carbon black in an 8:1:1 mass ratio in N-methylpyrrolidone (NMP) to produce a uniform slurry. The homogeneous slurry was blade-coated onto standard Celgard 2400 PP separators, which were later punched into 19 mm circular discs for cell assembly. The dried D-MIL-101 (Cr) coating layer shows a uniform thickness of ~9 μm from cross-sectional SEM characterization. Each blank 19 mm PP separator has a mass of 4 mg, and the dry coating mass loaded on each separator disc is 0.4 mg. The coating induces a 10% mass increment of the PP substrate, and the functional D-MIL-101 (Cr) coating accounts for ~9.09 wt% of the total mass of the composite separator.The sulfur cathode with an areal sulfur loading of 2 mg cm^{-2} was cut into circular discs, and full coin cells were assembled inside an argon-filled glove box. Each cell was injected with 40 μL electrolyte, giving an electrolyte/sulfur (E/S) ratio of 20 μL mg^{-2}. A 16 mm-diameter lithium foil with a thickness of 200 μm served as the metal anode, paired with 19 mm-diameter separators.

Material Characterization

Crystallinity was analyzed by powder X-ray diffraction (XRD) using a Bruker D8 ADVANCE X-ray diffractometer (with Cu Kα radiation). Pore volume and specific surface area were determined from N_2 adsorption-desorption isotherms measured at 77 K using a Micromeritics ASAP 2460 analyzer; The specific surface area was calculated using the Brunauer-Emmett-Teller (BET) model. The chemical structure was verified by Fourier-transform infrared spectroscopy (FT-IR, Bruker VERTEX 70) and Raman spectroscopy (Thermo Scientific, Waltham, MA). Morphological features were characterized using field-emission scanning electron microscopy (SEM, Zeiss

Gemini SEM 500). Wettability was evaluated using dynamic contact angle measurements (Krüss DSA100). Zeta potential measurements (Zetasizer Nano ZSE) were performed using the same electrolyte solvent dispersion scheme to ensure reliable relative comparisons among MOF materials. Depth profile analysis of the surface composition of the lithium foil anode was conducted using time-of-flight secondary ion mass spectrometry (TOF-SIMS).

Calculation Method

All density functional theory (DFT) calculations were performed using the Gaussian 16 software package. An acyclic cluster model was employed to describe the interactions between MIL-101(Cr), D-MIL-101(Cr), and lithium polysulfides (Li_2S_x). Given the open-shell electronic structure of the Cr-based nodes, the relevant systems were treated using a spin-unrestricted approach. The geometries were optimized using the PBE0 hybrid functional combined with the def2-SVP basis set, and Grimme D3 dispersion correction and the Becke-Johnson damping function [PBE0-D3(BJ)] were introduced to describe the dispersion interactions in the system. DFT calculations employed an ultrafine numerical integration grid and strict SCF convergence criteria. For different Li_2S_x adsorption systems, initial adsorption configurations were constructed at representative potential adsorption sites on the MOF surface, followed by geometric optimization. By comparing the stability of different adsorption configurations, the most stable configuration with the lowest energy was selected as the optimal adsorption site and used for subsequent adsorption energy calculations. Based on the optimized structures, single-point energy calculations were further performed at the PBE0-D3(BJ)/def2-TZVP level; specifically, a hierarchical calculation strategy of PBE0-D3(BJ)/def2-TZVP//PBE0-D3(BJ)/def2-SVP was employed to obtain the electronic energies required for adsorption energy analysis. The adsorption energies were calculated as follows

$E_{ads} = E_{Li2Sx/sub} - E_{Li2Sx} - E_{sub}$

Where $E_{Li2Sx/sub}$, E_{Li2Sx} and E_{sub} are the total energies of the optimized adsorbate/substrate system, the Li_2S_x (S_8, Li_2S_8, Li_2S_6, Li_2S_4, Li_2S_2, and Li_2S) in the structure, and the clean substrate, respectively. This calculation does not include

explicit DME/DOL solvent molecules. To balance computational costs, an implicit continuous solvent model (SMD) is used to approximate the overall polarization environment of the electrolyte, which can, to some extent, reproduce the macroscopic polarization effects of the solvent surrounding the adsorbed polysulfides.

LiPS Adsorption Test

A 5 mmol L^{-1} solution of Li_2S_6 was prepared by mixing sulfur and Li_2S in the electrolyte in a stoichiometric ratio. This electrolyte was prepared by dissolving 1 M lithium bis(trifluoromethanesulfonyl)imide (LiTFSI) in a 1:1 volume mixture of 1,2-dimethoxyethane (DME) and 1,3-dioxolane (DOL), with the addition of 1% $LiNO_3$. Subsequently, 5 mg of MIL-101(Cr) and D-MIL-101(Cr) were added to the Li_2S_6 solution for 12 hours. The supernatant was collected and analyzed by UV-visible spectroscopy.

Li_2S Reduction Test

The electrolyte used was a 0.25 M Li_2S_8 solution; lithium foil was used as the anode and carbon cloth as the cathode, and the cells were assembled using different separators. The assembled button cells were discharged at a constant current of 0.112 mA until the voltage reached 2.06 V, and then discharged further at 2.05 V until the current dropped below 10^{-5} A.

Symmetric Cell Testing

In the symmetric cell configuration, MIL-101(Cr) and D-MIL-101(Cr) were coated onto aluminum foil and cut into 12-mm circular discs, which served as both the working electrode and the counter electrode. The symmetric cells were characterized using an electrolyte containing 0.1 M Li_2S_6. Cyclic voltammetry (CV) measurements were performed over a potential range of -1 to 1 V at a scan rate of 10 mV s^{-1} (using the Chenhua CHI-660C electrochemical workstation). Electrochemical impedance spectroscopy (EIS) was recorded over a frequency range of 100 kHz to 0.1 Hz.

Lithium-Sulfur Battery Assembly Process

First, the prepared coated aluminum foil is cut into 12-mm circular discs to serve as the cathode. The lithium-sulfur batteries are then assembled in an argon-filled glove box. During assembly, a sulfur/carbon composite material is used for the cathode, 16-

mm lithium foil for the anode, and both 19-mm modified separators and conventional separators are employed. The electrolyte consists of 1 M LiTFSI dissolved in a mixed solvent of 1,2-dimethyl ether and 1,3-diethanolamine (1:1 by volume), with 1% $LiNO_3$ added. Cyclic voltammetry (CV) curves in the voltage range of 1.6–2.8 V and electrochemical impedance spectra (EIS) in the frequency range of 100 kHz to 0.1 Hz were obtained using an electrochemical workstation (Chenhua CHI-660C). For a standard sulfur electrode (approximately 2 mg cm^{-2}), the mass ratio of electrolyte to sulfur (E/S) was 20 μL mg^{-1}; meanwhile, the high-loading electrode (4 mg cm^{-2}) exhibited a lower E/S ratio (10 μL mg^{-1}). The battery's cycling performance and rate capability were evaluated at 25°C using a Neware battery testing system, with a voltage range of 1.6–2.8 V.

Lithium Ion Mobility (DC Polarization—Bruce-Vincent Method)

Lithium ion mobility refers to the proportion of lithium ions in the total ionic migration (in this experiment, lithium ions and anions in the electrolyte). It reflects the separator's selective transport capacity for lithium ions. It is measured and calculated using the DC polarization method, with the following formula:

$tLi+ = I_{ss} \cdot R_0 / R_{ss} \cdot I_0$

I_0: Initial current (current at the moment of polarization)

I_{ss}: Steady-state current (current after polarization reaches steady state)

R_0: Total resistance of the cell before polarization (obtained from AC impedance (EIS) testing)

R_{ss}: Total resistance of the cell after polarization (obtained from AC impedance (EIS) testing)

Ionic Conductivity (EIS AC Impedance Method)

Ionic conductivity is a key parameter for measuring a separator's ability to allow ions to pass through; it reflects the efficiency of ion transport within the separator. It is calculated using impedance data obtained from an AC impedance spectroscopy (EIS) test, according to the following formula:

$\sigma = d/R_b \cdot S$

σ: Ionic conductivity, units S cm^{-1}

d: Membrane thickness, unit: cm

R_b: Membrane intrinsic impedance (intercept on the real axis in the high-frequency region of the EIS plot), unit: Ω

S: Effective electrode contact area, unit: cm^2

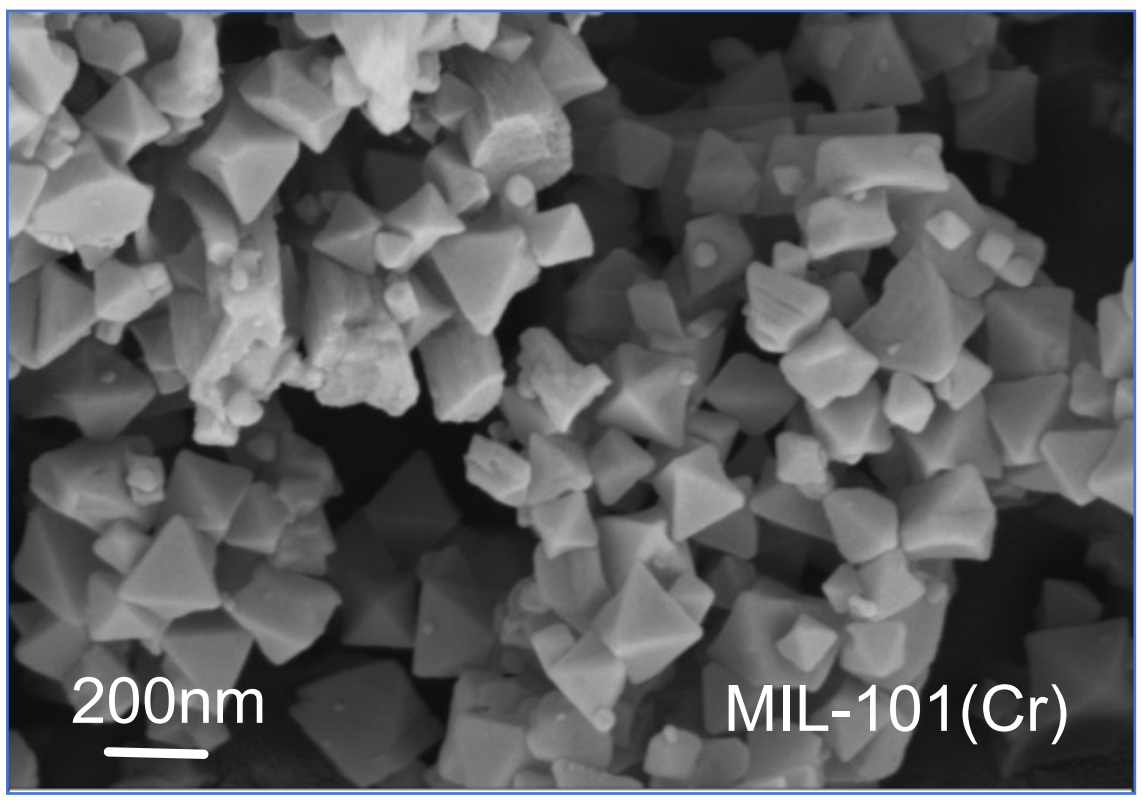

Fig. S1 Scanning electron microscopy (SEM) image of MIL-101(Cr)

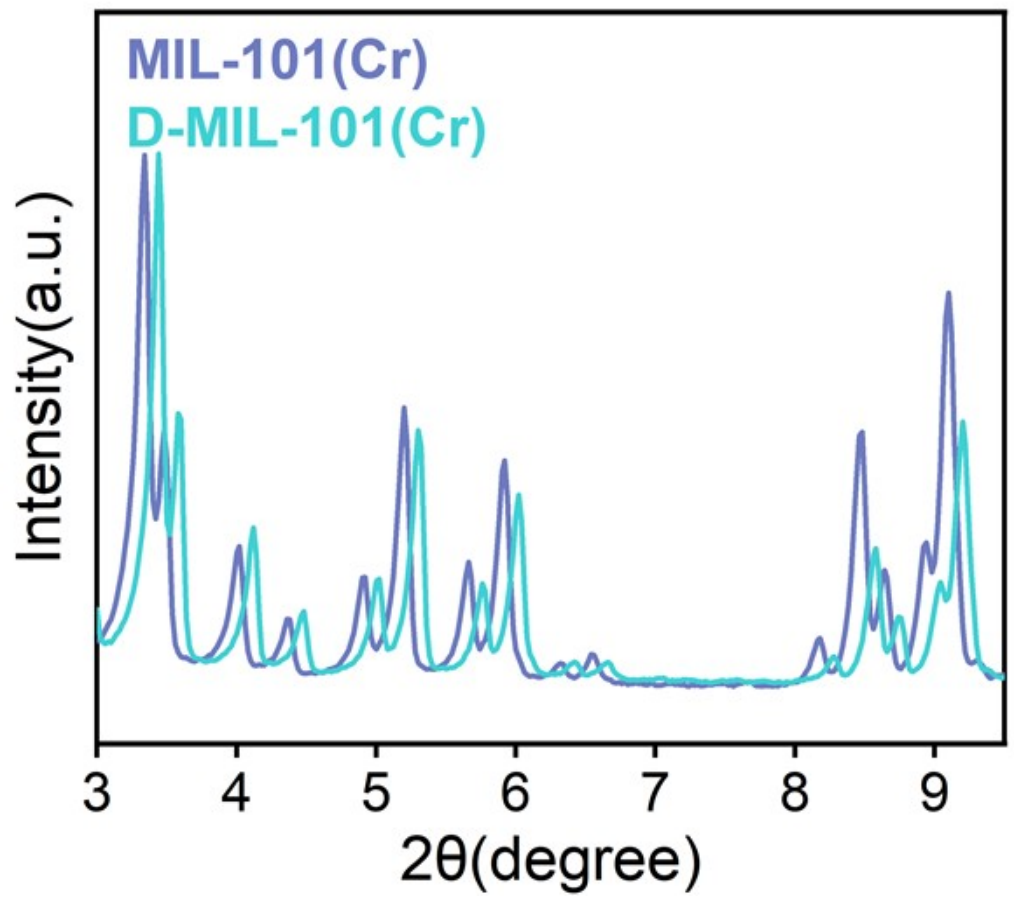

Fig. S2 XRD patterns of MIL-101(Cr) and D-MIL-101(Cr)

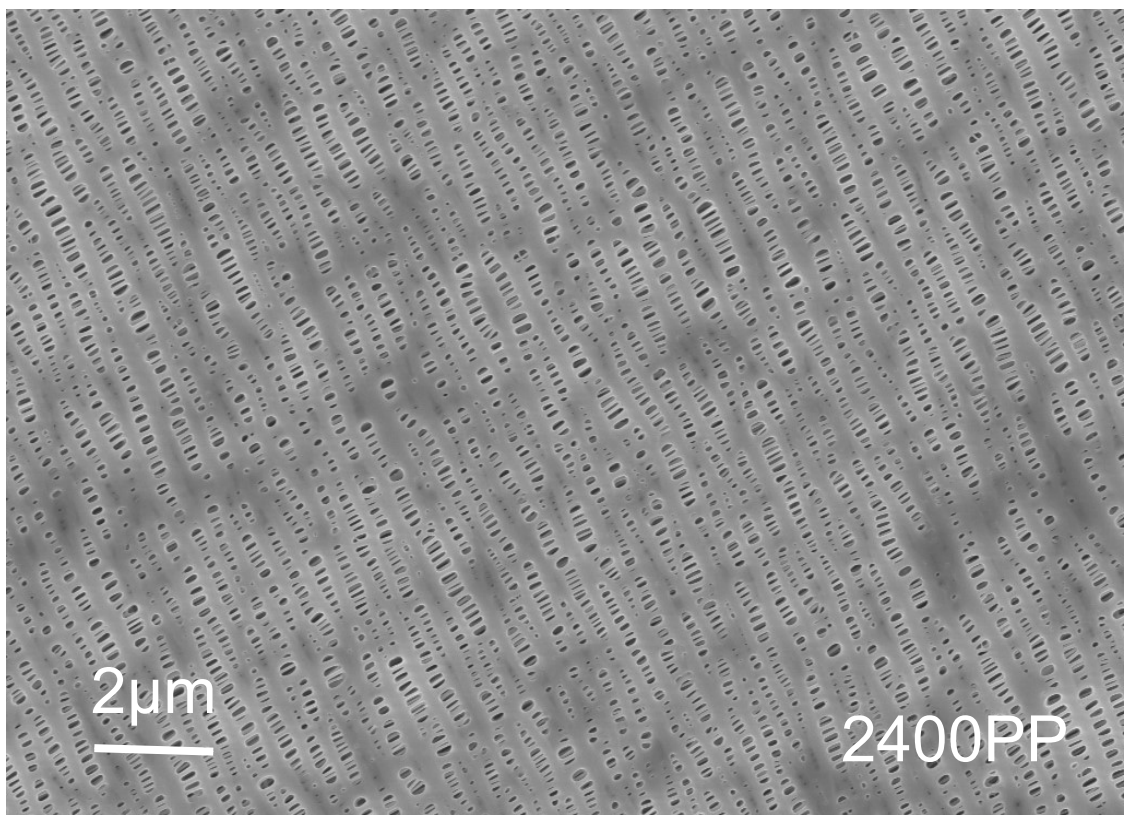

Fig. S3 SEM image of the 2400PP membrane surface

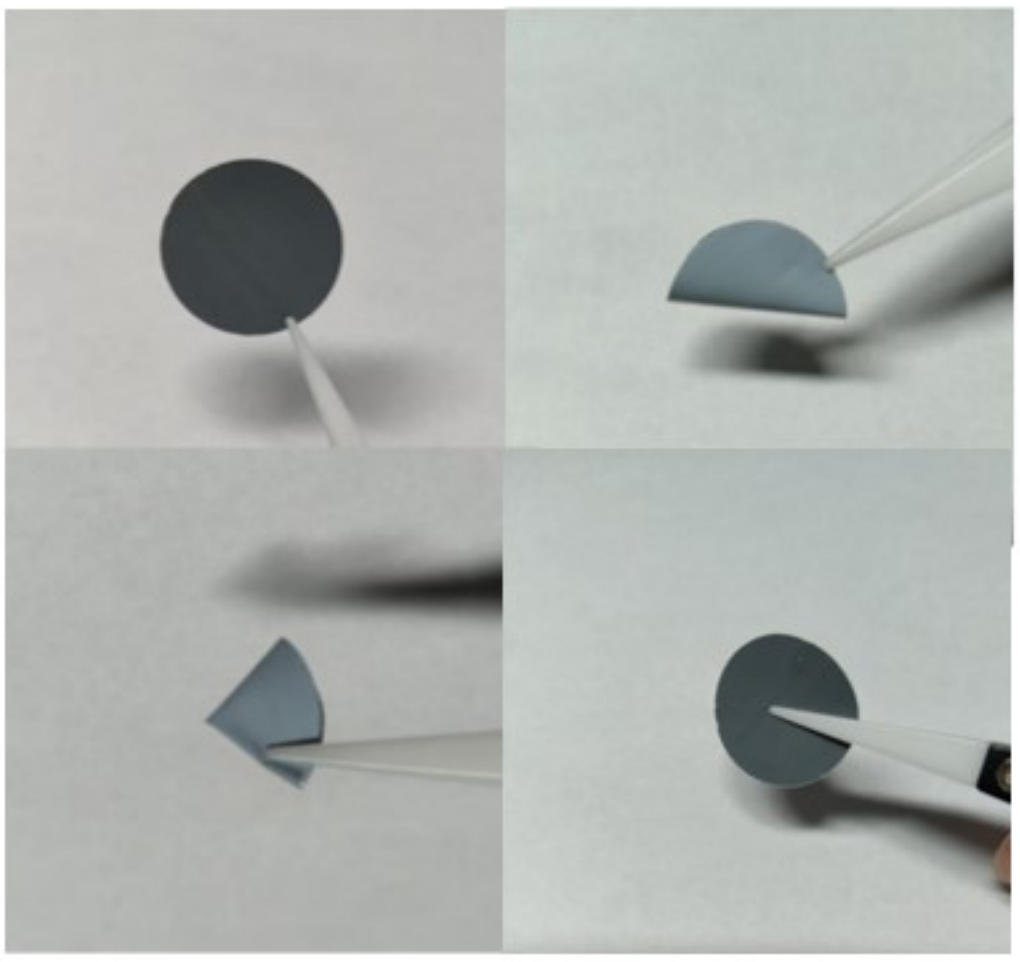

Fig. S4 D-MIL-101(Cr)/PP modified separators folded image

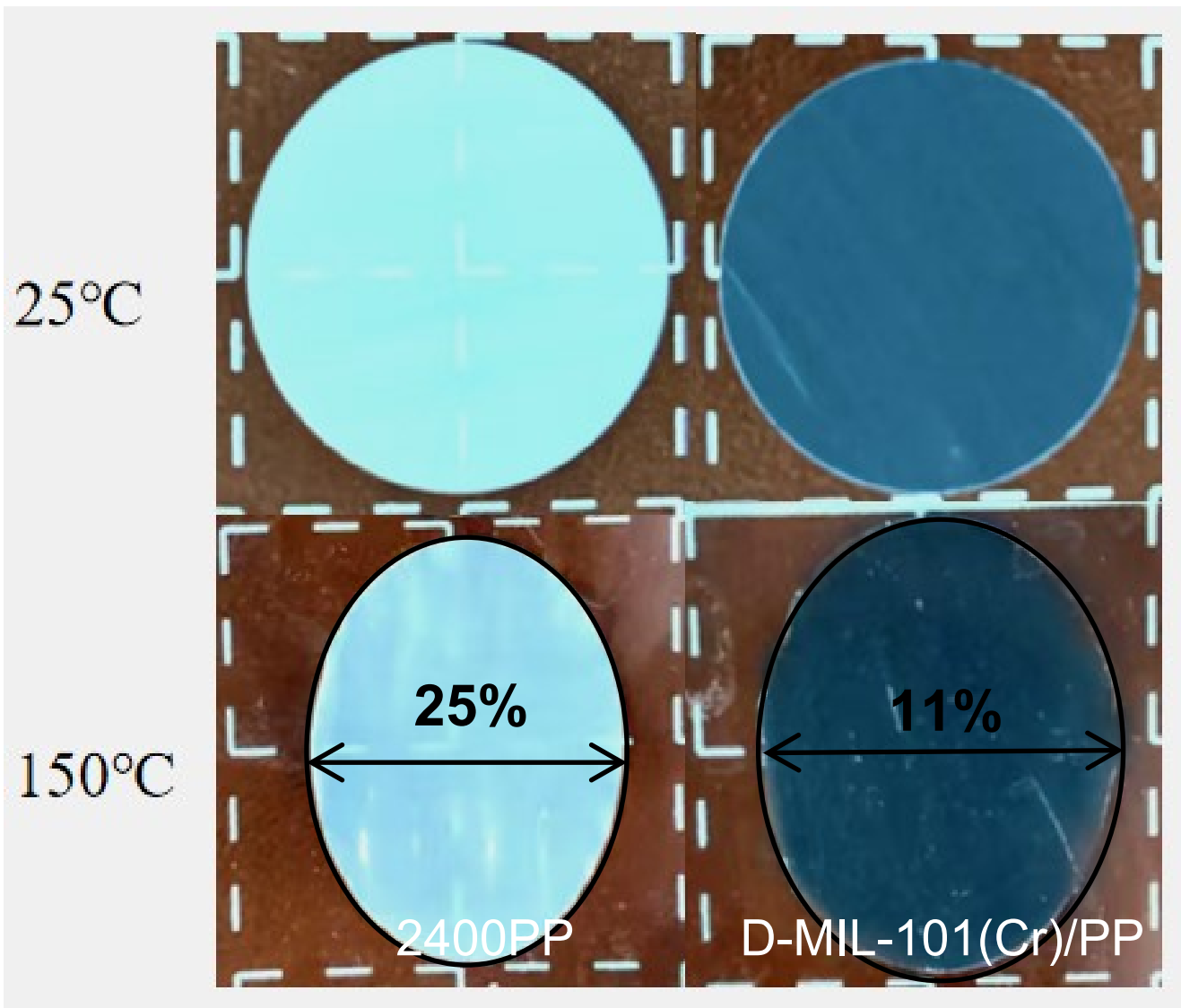

Fig. S5 Thermal stability test results for D-MIL-101(Cr)/PP modified separators and PP separators

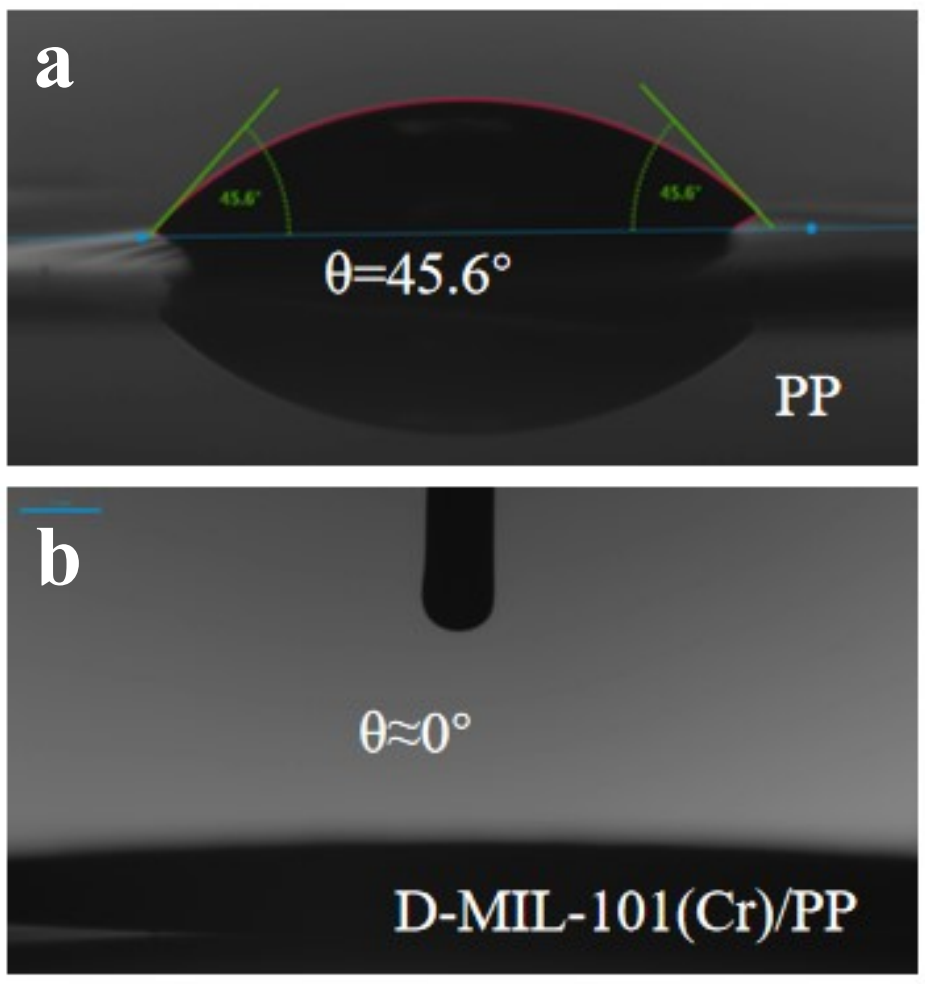

Fig. S6 Contact angle testing of (a) 2400PP and (b) D-MIL-101(Cr)/PP modified separators

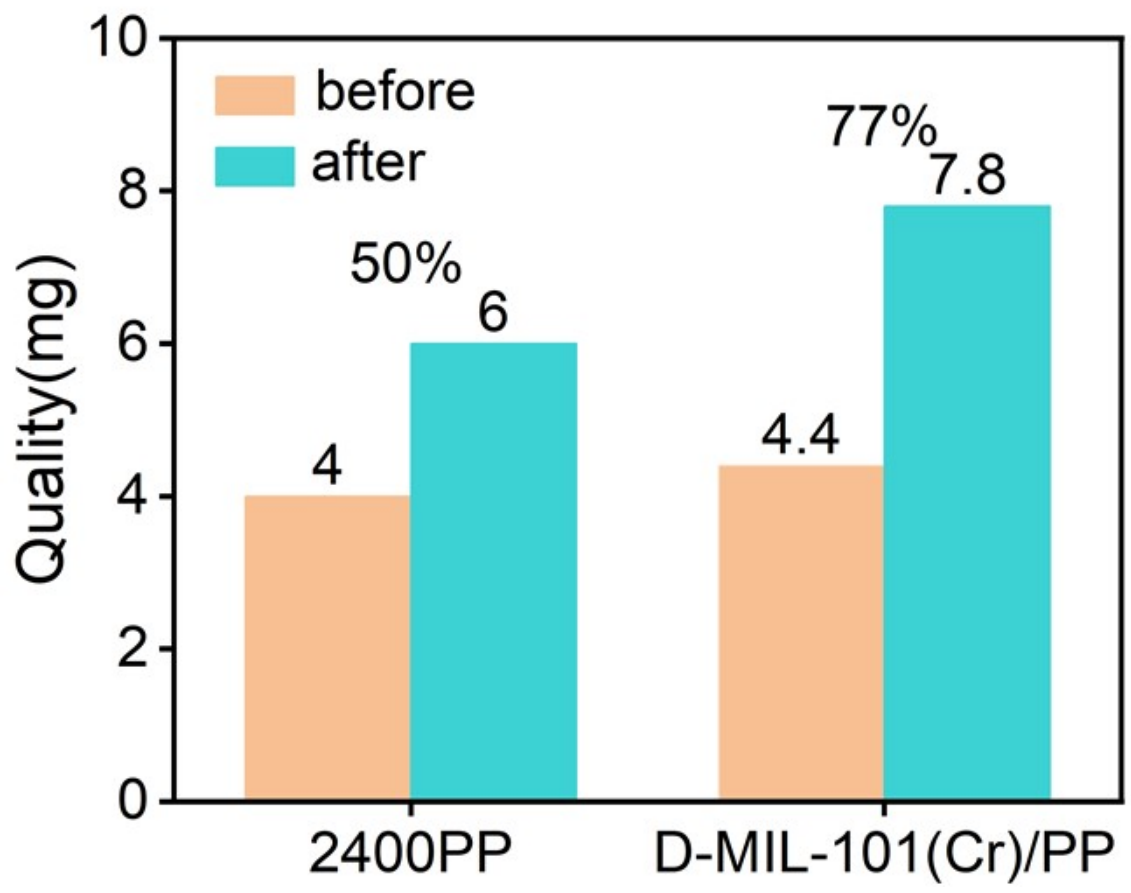

Fig. S7 Lithium-sulfur electrolyte absorption rates of D-MIL-101(Cr)/PP modified separators and PP separators

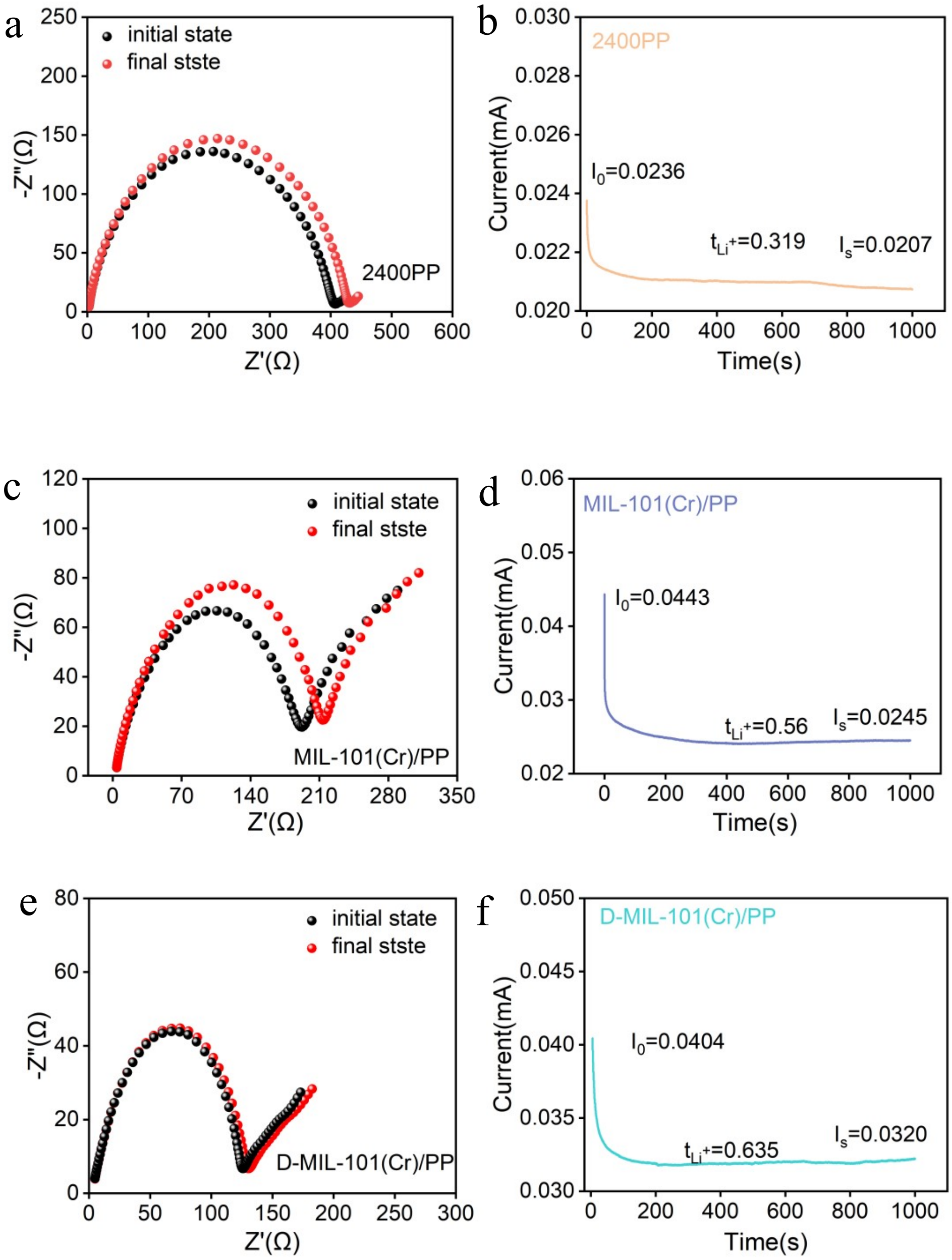

Fig. S8 (a)-(f) Instantaneous current testing of lithium-lithium symmetric batteries with different separators

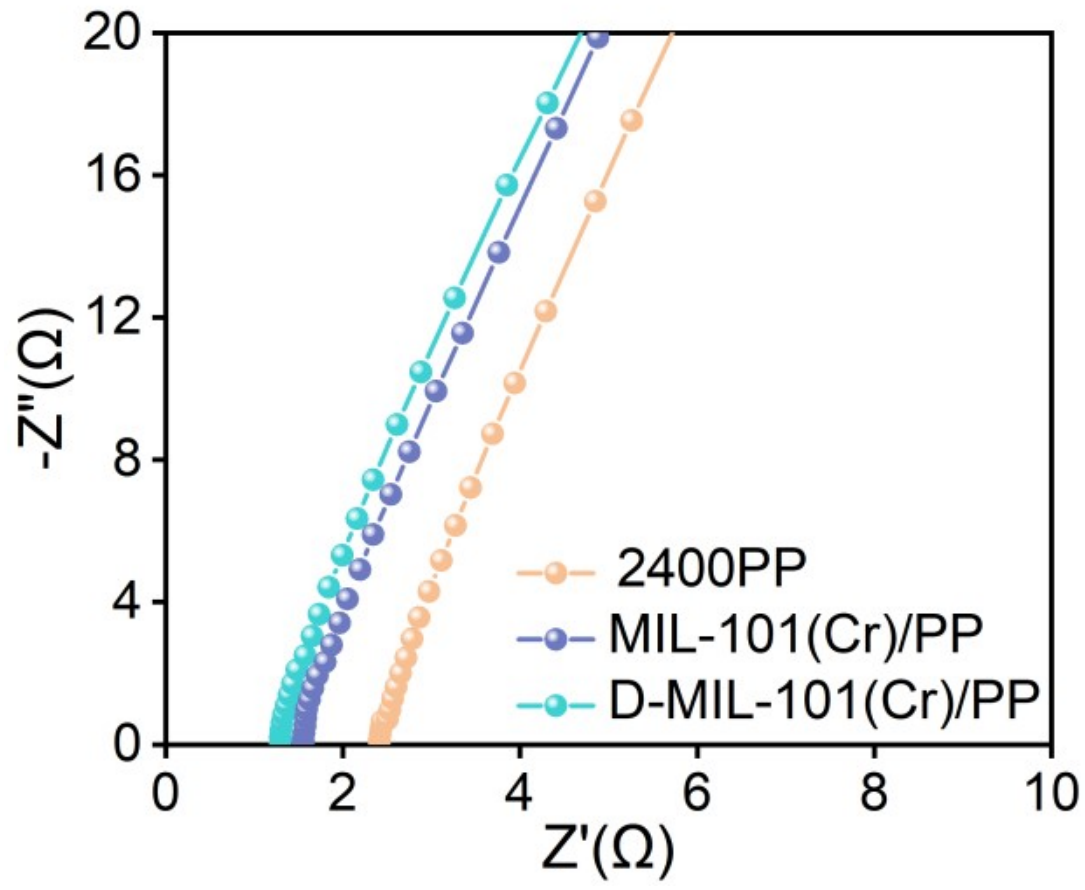

Fig. S9 Impedance of symmetrical stainless steel cells with different separators

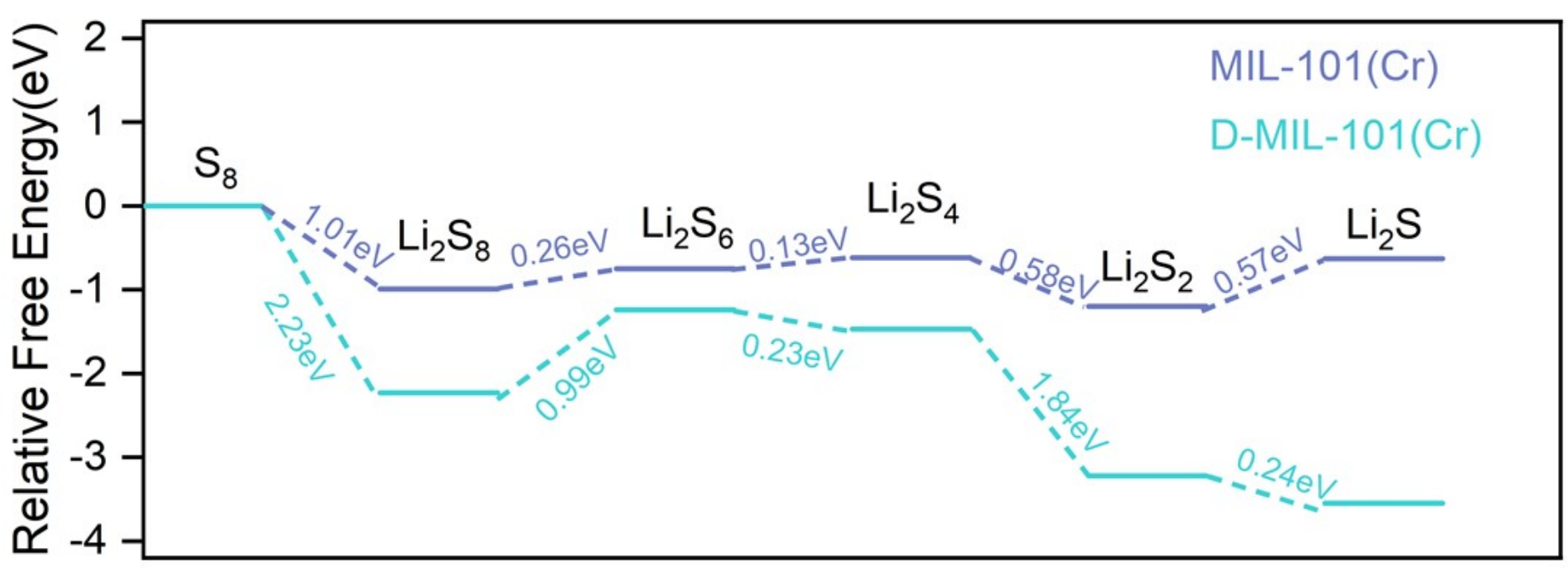

Fig. S10 Catalytic conversion capabilities of MIL-101(Cr) and D-MIL-101(Cr) for different polysulfides

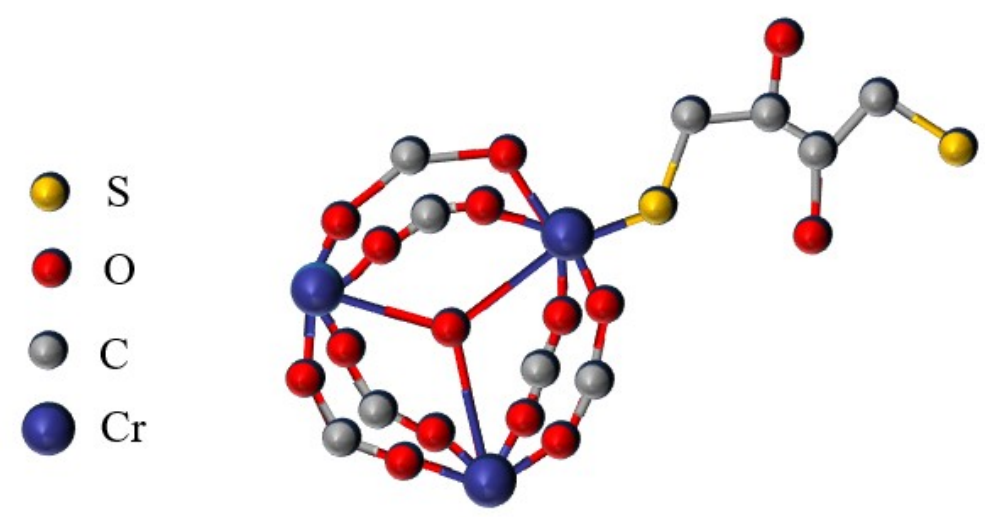

Fig. S11 Schematic diagram of the D-MIL-101(Cr) ball-and-rod model

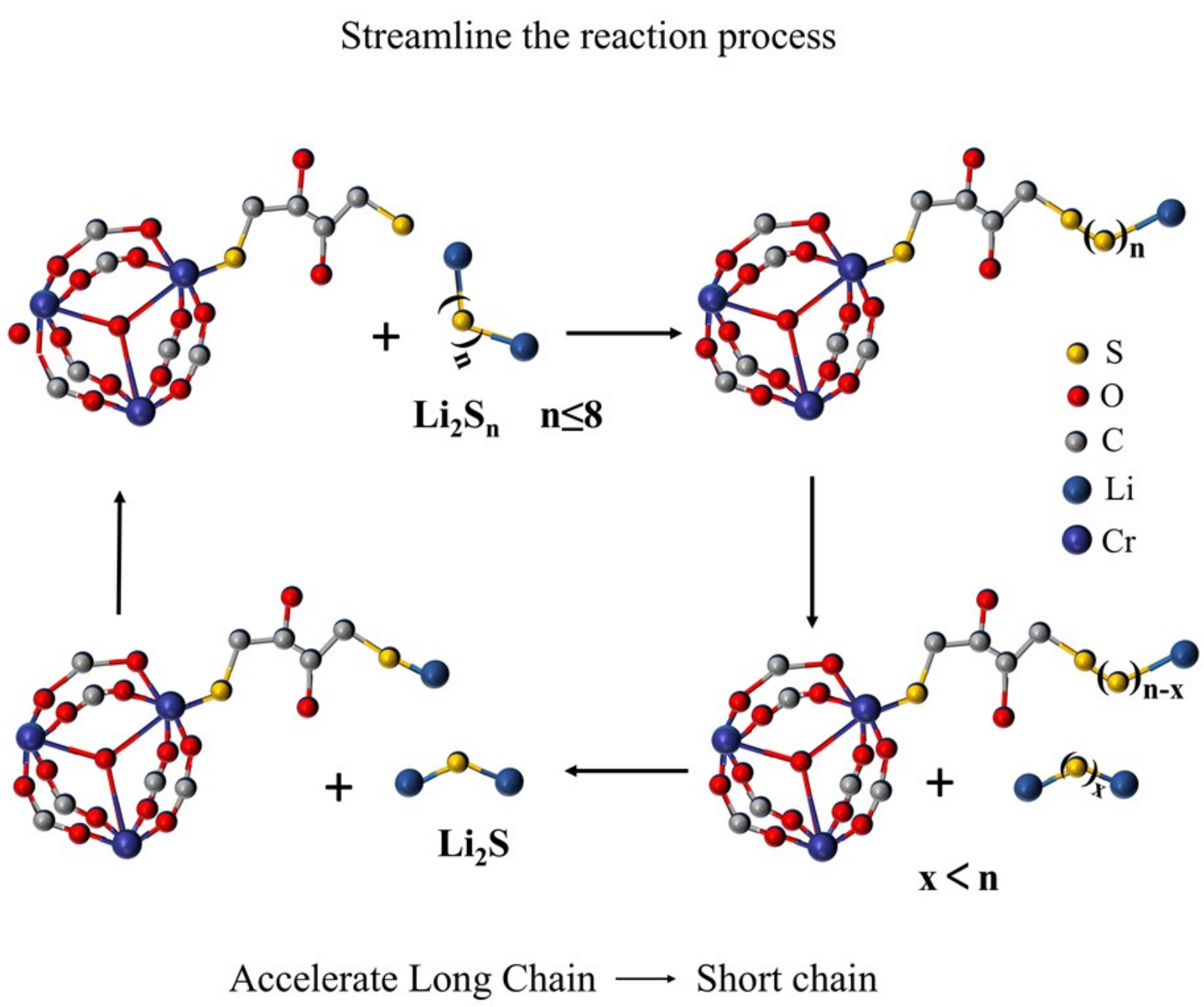

Fig. S12 Schematic diagram of simplified redox reaction pathways

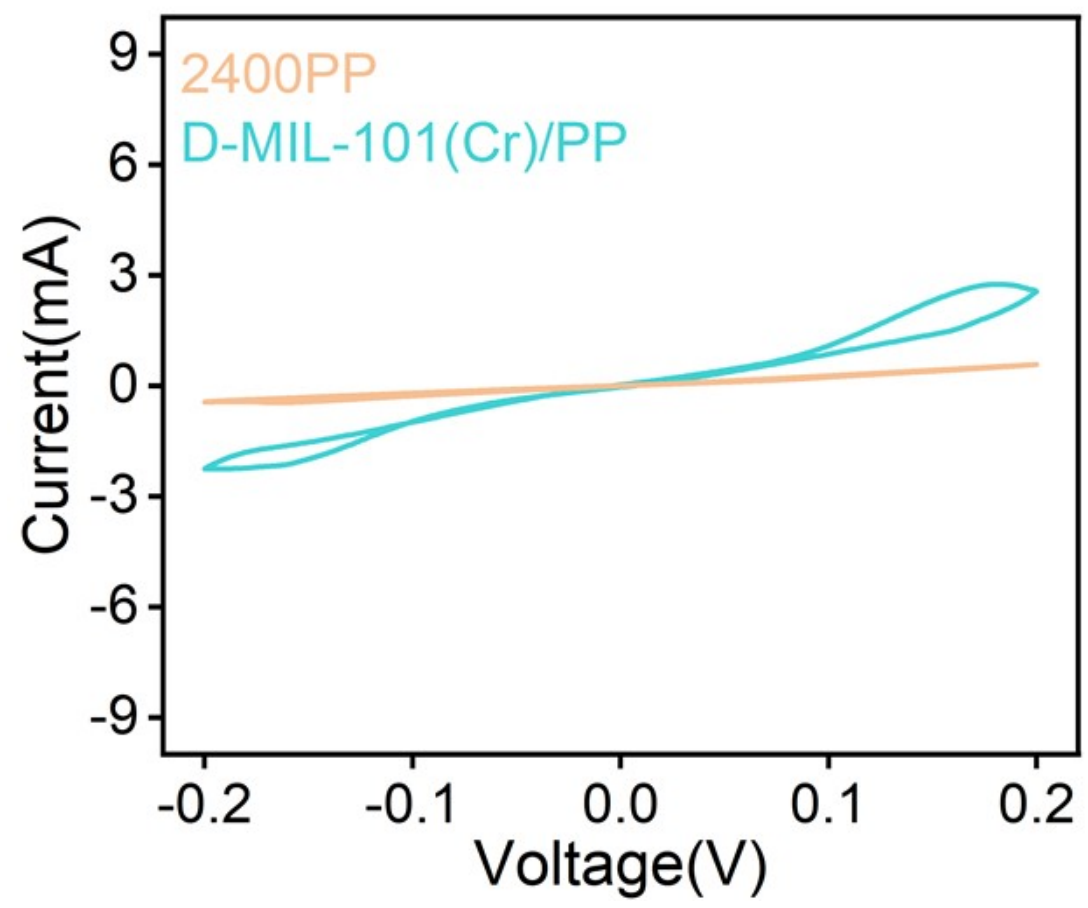

Fig. S13 CV Testing of lithium-Lithium symmetric cells with different separators

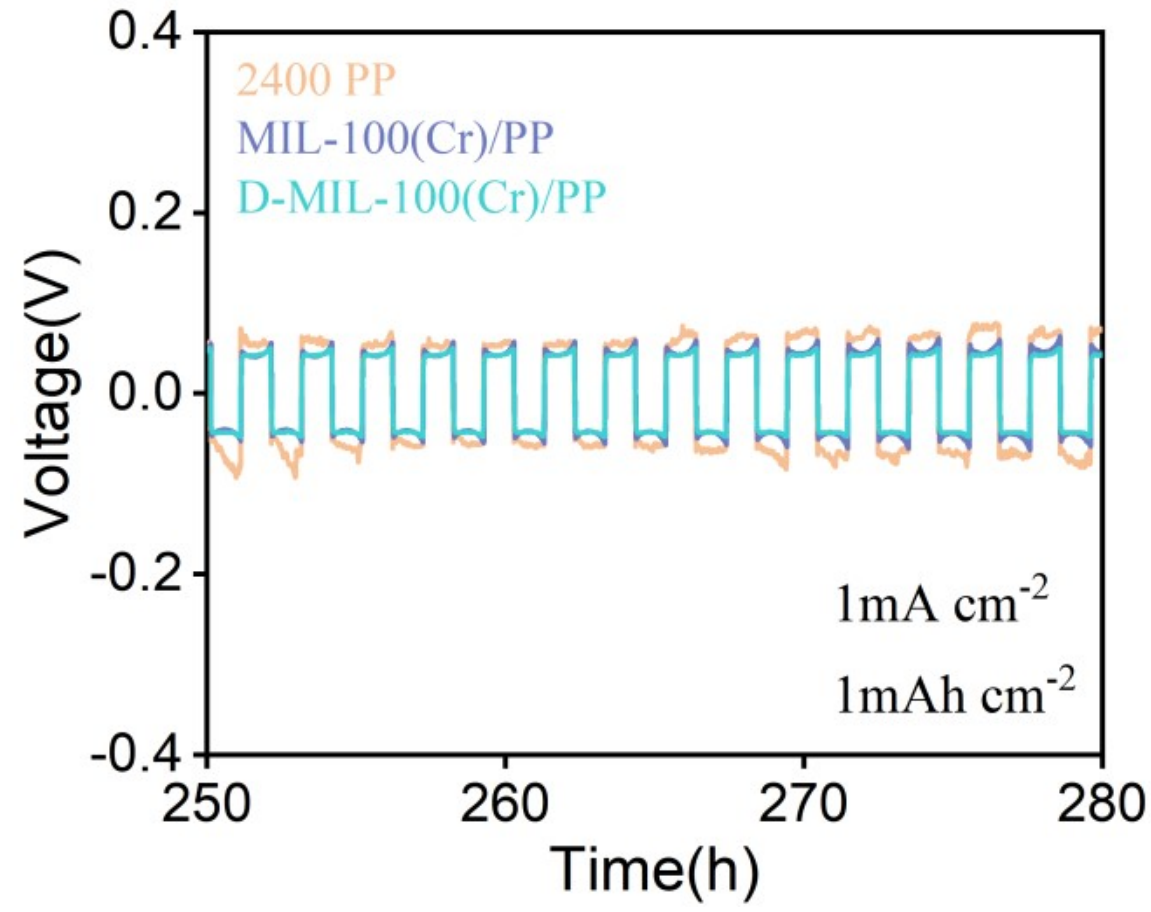

Fig. S14 Long-Cycle testing of lithium-lithium symmetric batteries with different separators

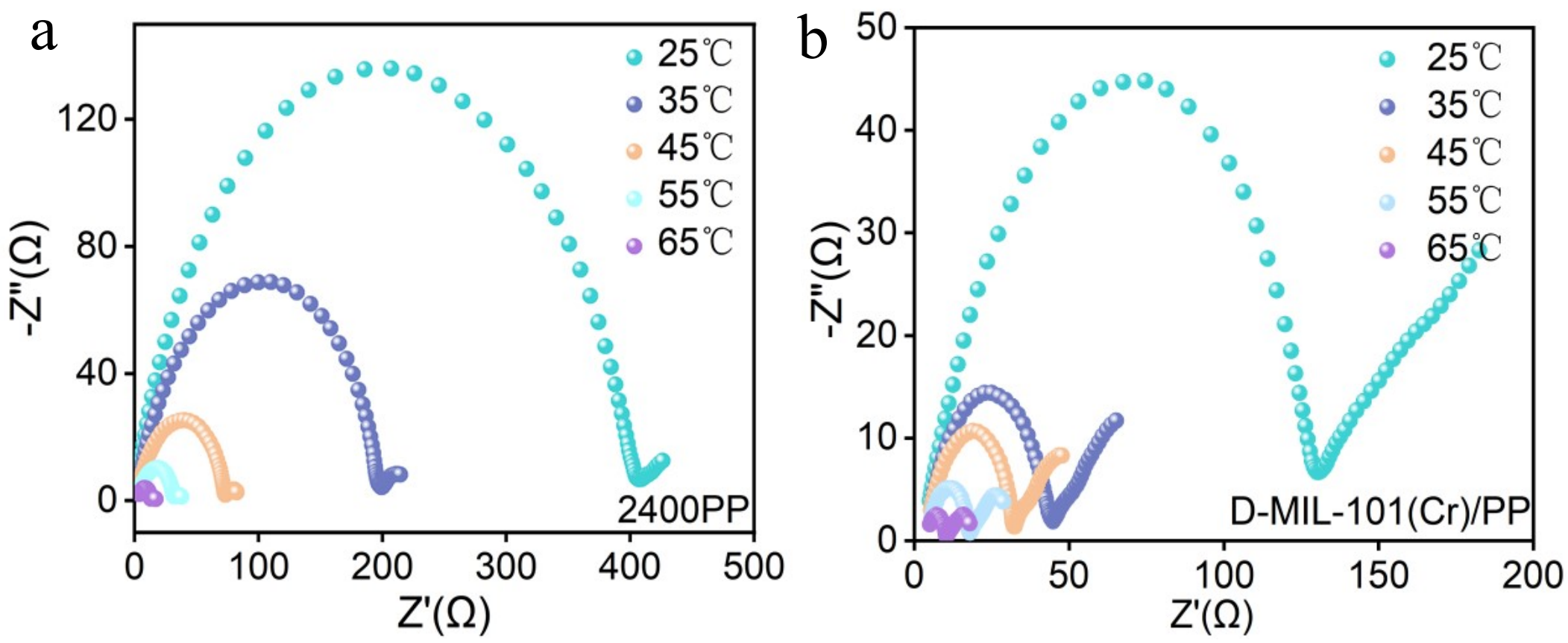

Fig. S15 Impedance of lithium-lithium symmetric batteries with D-MIL-101(Cr)/PP (b) and PP-(a) separator at different temperatures

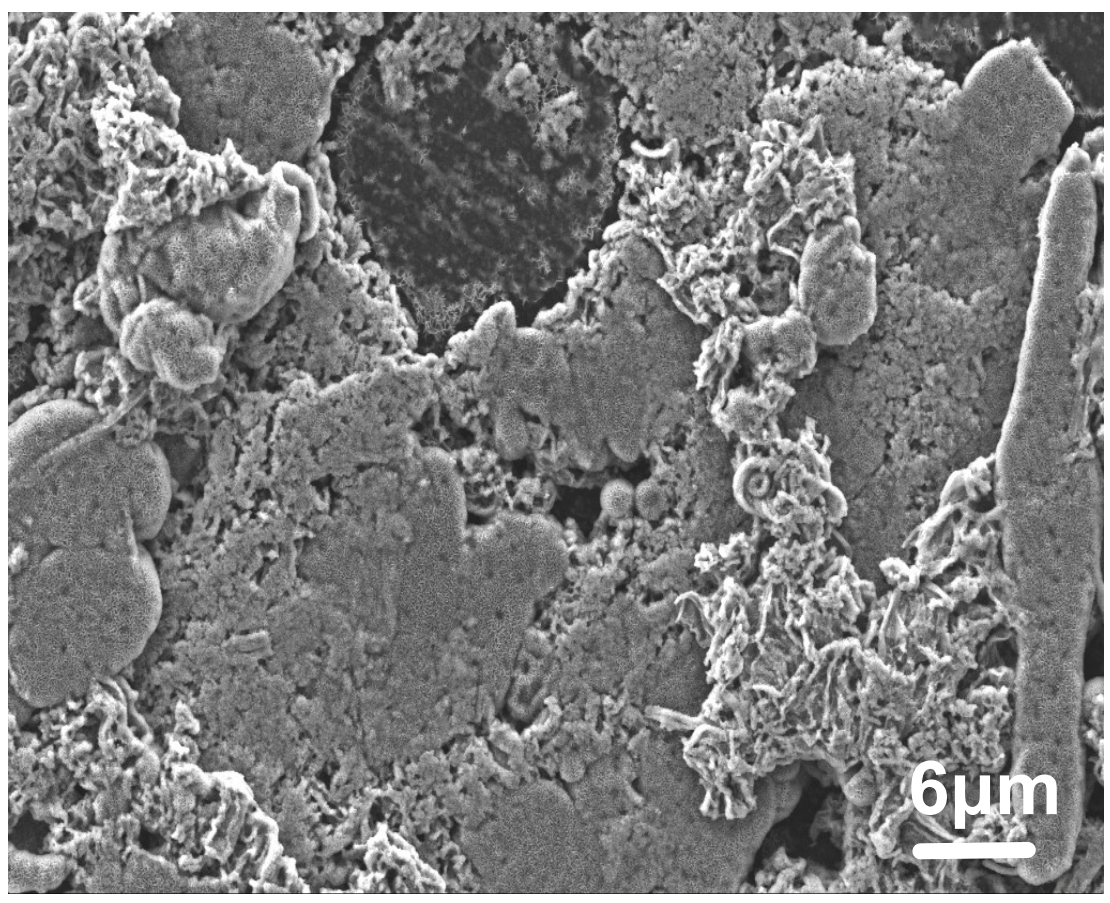

Fig. S16 Surface morphology of lithium metal after cycling of lithium-lithium symmetric cells with PPseparator

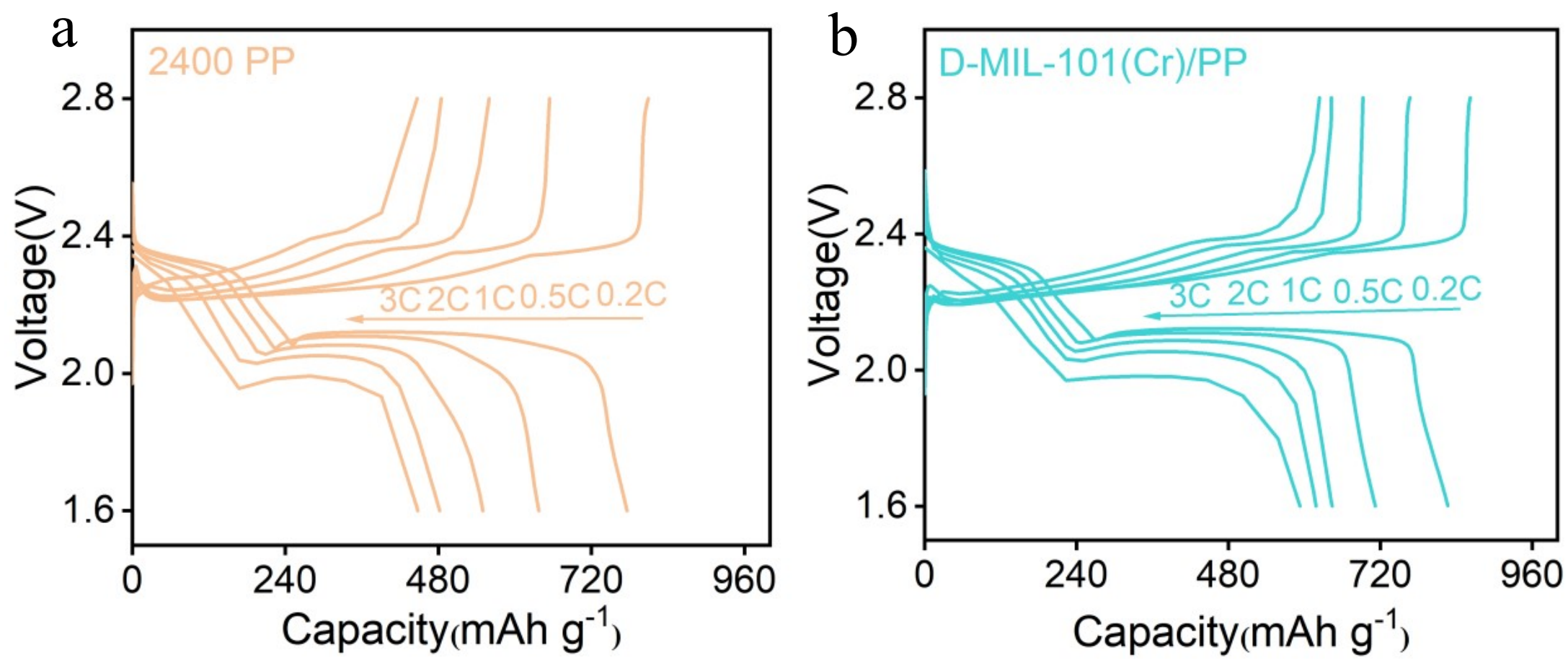

Fig. S17 Rate-test charge-discharge curves for lithium-sulfur batteries with D-MIL-101(Cr)/PP (b) and PP-(a) separator

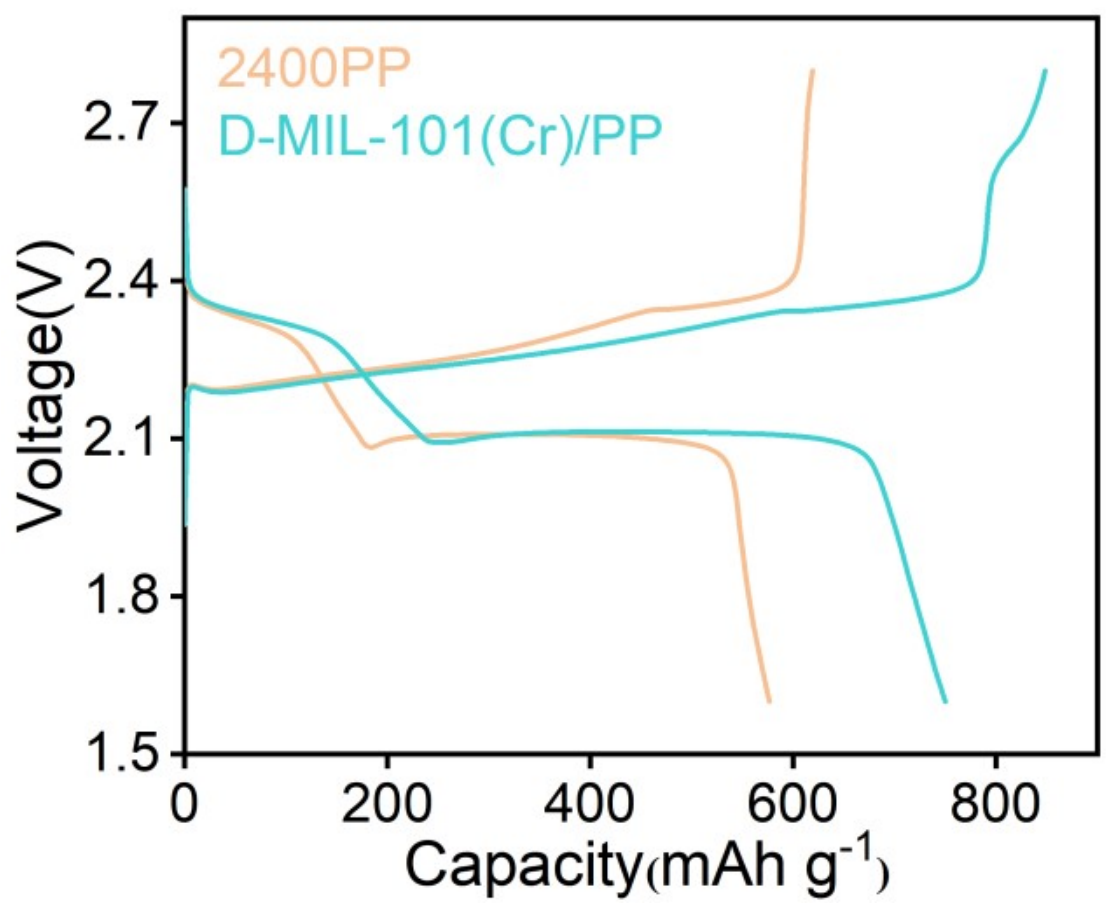

Fig. S18 Charge-discharge curves of lithium-sulfur batteries with D-MIL-101(Cr)/PP and PP separator

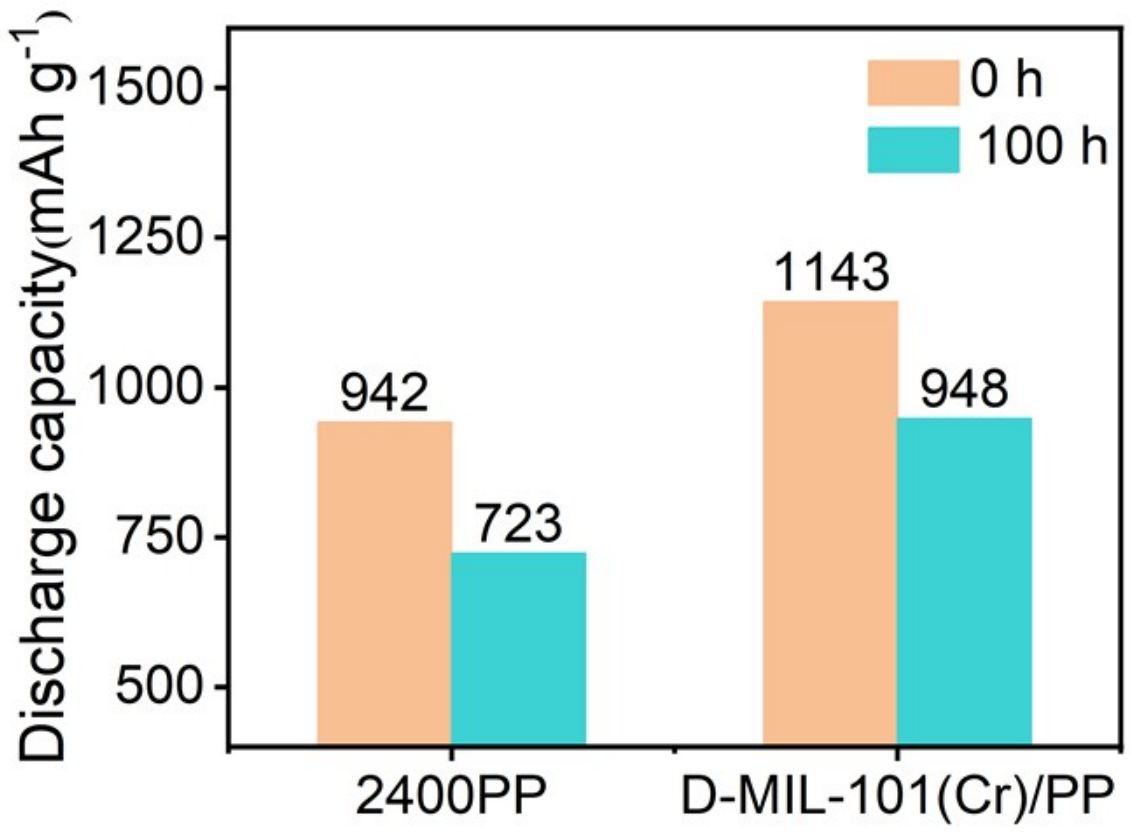

Fig. S19 Self-discharge capacity testing of lithium-sulfur batteries with D-MIL-101(Cr)/PP and PP separator

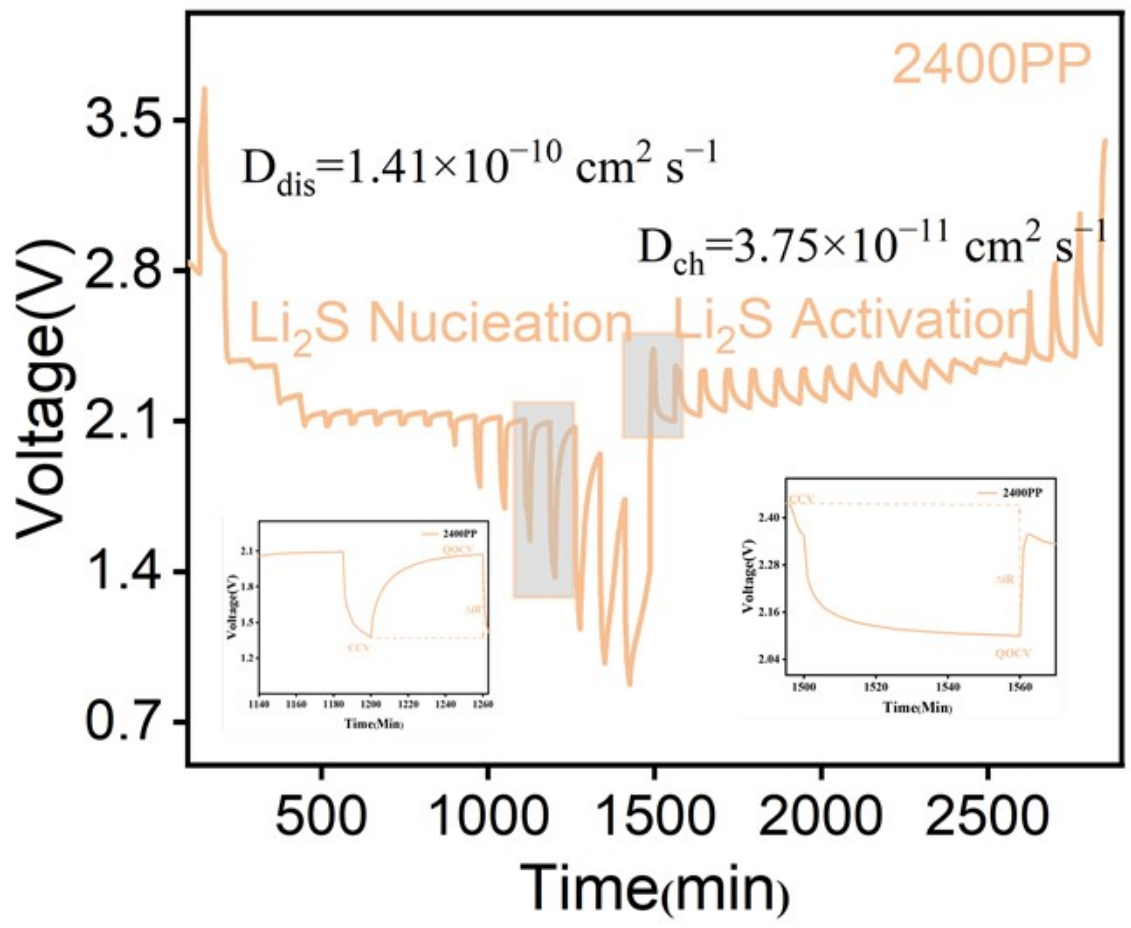

Fig. S20 GITT curve for lithium-sulfur batteries with PP separator

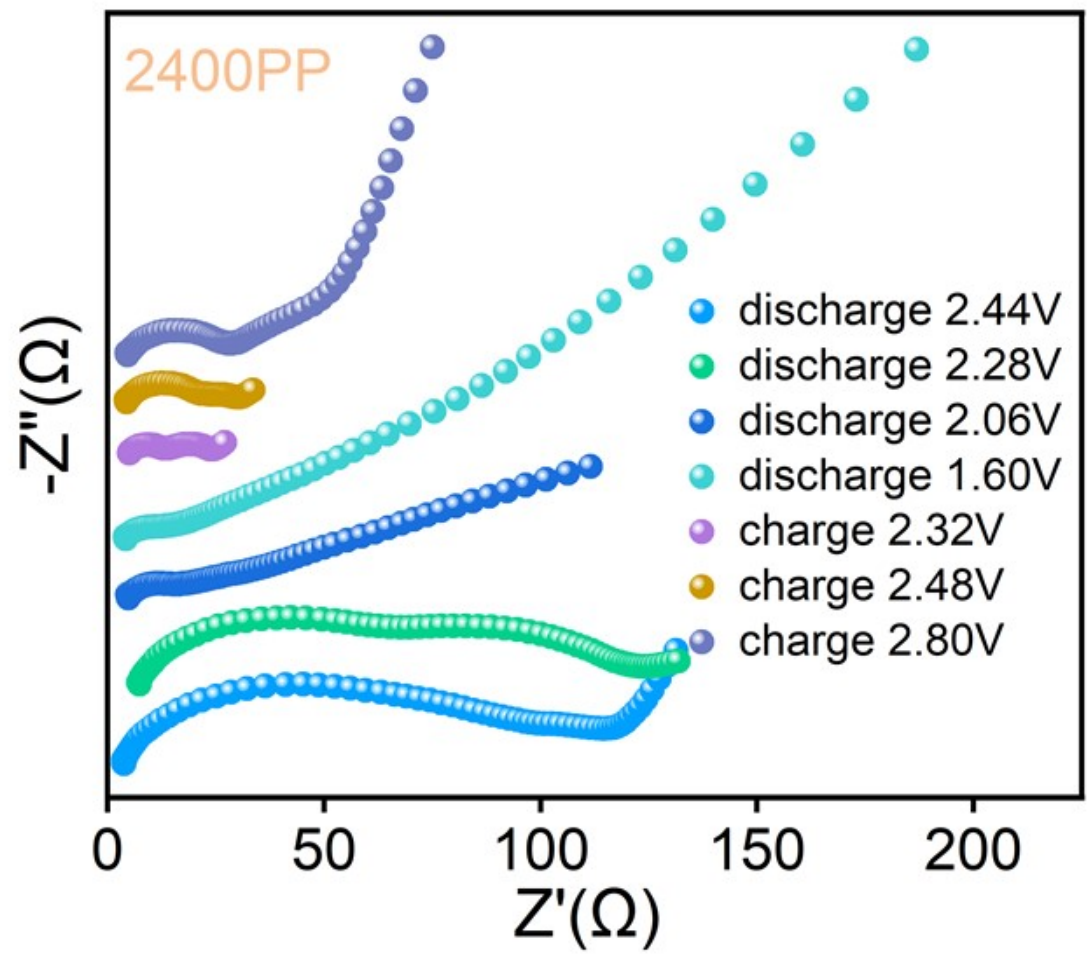

Fig. S21 In-situ EIS of lithium-sulfur batteries with PP separator

Table S1. EDS Elemental Composition Map

Elements	Line Type	Apparent concentration	k-ratio	Wt%	Wt% Sigma	At%	Standard Sample Label	Manufacturer Standards
C	Candlestick Charts	100.17	1.00165	85.33	0.94	92.44	C Vit	Yes
O	Candlestick Charts	10.64	0.03579	6.44	0.16	5.24	SiO2	Yes
S	Candlestick Charts	4.02	0.03463	1.67	0.08	0.68	FeS2	Yes
Cr	L Series	4.30	0.04297	6.56	1.02	1.64	Cr	Yes
Total：				100.00		100.00		
